# Relationship between risk, cumulative burden of exacerbations and mortality in patients with COPD: modelling analysis using data from the ETHOS study

**DOI:** 10.1186/s12874-022-01616-7

**Published:** 2022-05-25

**Authors:** Kirsty Rhodes, Martin Jenkins, Enrico de Nigris, Magnus Aurivillius, Mario Ouwens

**Affiliations:** 1 Medical & Payer Evidence Statistics, Real-World Science and Digital, BioPharmaceuticals Medical Evidence, AstraZeneca, Academy House, Hills Road, Cambridge, CB2 8PA UK; 2 BioPharmaceuticals R&D, AstraZeneca, Cambridge, UK; 3 Formerly of AstraZeneca, Cambridge, UK; 4 BioPharmaceuticals R&D, AstraZeneca, Gothenburg, Sweden; 5 Medical & Payer Evidence Statistics, Real-World Science and Digital, BioPharmaceuticals Medical Evidence, AstraZeneca, Gothenburg, Sweden

**Keywords:** Chronic obstructive pulmonary disease, Disease progression, Cost of illness, Incidence

## Abstract

**Background:**

The major drivers of cost-effectiveness for chronic obstructive pulmonary disease (COPD) therapies are the occurrence of exacerbations and deaths. Exacerbations, including acute and long-term events, can cause worsening of COPD and lead to an increased risk of further exacerbations, and ultimately may elevate the risk of death. In contrast to this, health economic models are based on COPD severity progression. In this post hoc analysis of the ETHOS study, we focus on the progression of COPD due to exacerbations and deaths.

**Methods:**

We fitted semi-parametric and fully parametric multi-state Markov models with the following five progressive states: State 1, no exacerbation; State 2, 1 moderate exacerbation; State 3, ≥ 2 moderate exacerbations; State 4, ≥ 1 severe exacerbations; State 5, death. The models only allowed a patient to transition to a worsened health state, and transitions did not necessarily have to be to the next adjacent state. We used the multi-state models to analyse data from ETHOS, a phase III, 52-week study assessing the efficacy and safety of triple therapy with budesonide/glycopyrronium/formoterol fumarate dihydrate in moderate-to-very severe COPD.

**Results:**

The Weibull multi-state Markov model showed good fit of the data. In line with clinical evidence, we found a higher mortality risk after a severe exacerbation (11.4-fold relative ratio increase [95% CI, 7.7–17.0], 6.4-fold increase [95% CI, 3.8–10.8] and 5.4-fold increase [95% CI, 2.9–10.3] relative to no exacerbations, 1 moderate exacerbation or ≥ 2 moderate exacerbations, respectively). One moderate exacerbation increased mortality risk 1.8-fold (95% CI, 1.1–2.9) vs no exacerbations. We also found a higher risk of severe exacerbation and mortality following ≥ 2 moderate exacerbations.

**Conclusion:**

Multi-state modelling of patients with COPD in ETHOS found an acute and chronic effect of severe exacerbations on mortality risk. Risk was also increased after a moderate exacerbation. Clinical management with effective pharmacotherapies should be optimised to avoid even moderate exacerbations. Modelling with exacerbations could be an alternative to current COPD models focused on disease progression.

**Trial registration:**

NCT02465567

**Supplementary Information:**

The online version contains supplementary material available at 10.1186/s12874-022-01616-7.

## Background

Chronic obstructive pulmonary disease (COPD) is the third-leading cause of death worldwide [1]. Globally, it is estimated that more than 300 million people have COPD [2], but over half of these cases may be undiagnosed [3]. This high prevalence has been estimated to translate to over $50 billion per year of direct and indirect costs in the US alone [4], with the majority of costs attributed to COPD exacerbations [5, 6].

Exacerbations, defined as an acute worsening of respiratory symptoms that require additional therapy, are a hallmark of COPD. Exacerbations are classified as mild, moderate, or severe and, depending on severity, typically last for 7–10 days [4]. Mild exacerbations may be self-managed with an increase in short-acting rescue medication. Moderate exacerbations require treatment with short-acting rescue medication plus oral steroids and/or antibiotics [4, 7]. Severe exacerbations are distinguished by the requirement for treatment in the emergency room and/or hospitalisation and may lead to death [4, 7], and are, consequently, considerably more costly and resource-demanding than other exacerbations [8]. This is compounded by the high frequency of re-hospitalisation following discharge [9, 10]. Patients with frequent exacerbations endure more work absences and have more short-term disability days per year, corresponding to short-term disability costs approximately twice as high as patients with infrequent or no exacerbations [11]. Exacerbations also have a significant burden on caregivers and families [12, 13].

Patients at greatest risk of exacerbations are those who have a history of exacerbations, as well as those with high symptom burden and comorbid conditions [14–16]. In addition, the risk of severe exacerbation or death has been found to increase with each subsequent exacerbation [17]. Following a moderate exacerbation, while lung function declines more rapidly, [18] the risks of cardiovascular complications, such as myocardial infarction and stroke [19], have been shown to increase. Exacerbations have also been shown to have a negative impact on symptom duration, quality of life, physical activity, lung function, mortality and mental health, and are associated with a number of different comorbidities [20, 21].

Health economics analyses of COPD typically focus on progression to increasing COPD severities and how, as patients progress and forced expiratory volume in 1 s (FEV_1_) decreases, the risk of exacerbations increases. Progression is usually taken into consideration using Markov models (a type of multi-state model), where the risk of an outcome (e.g. exacerbation) depends only on current state (i.e. the COPD severity at a fixed point in time) and does not consider exacerbation history. However, patients with very severe COPD already have a very low FEV_1_ that may not decline further, and they continue to accumulate exacerbations more frequently than patients with less severe COPD, increasing the risk of subsequent severe exacerbations and death.

A previous study using Cox models, which are commonly used for time-to-event analyses [22], showed that risk of future severe exacerbations and death increases in a stepwise manner with each additional moderate exacerbation, and that severe exacerbations were associated with a higher risk of death than multiple moderate exacerbations [23]. In comparison with Cox models, which use intermediate events as time-dependent covariates, multi-state models allow the estimation of hazards of moving between pairs of events for different patient profiles over the study observation period in order to understand the occurrence of events as a function of the natural history of disease. This facilitates simultaneous prediction of time to next exacerbation and time to death and enables investigation of the impact of patient characteristics and disease history on these outcomes. Thus, through improved understanding of disease progression and informed assessment of the impact of the disease and the associated costs, this may enable the generation of new insights regarding future risk [24] and potential improvements in the clinical management of patients.

This analysis sought to determine whether multi-state modelling, in the form of a Markov model, could be used to quantify how the accumulation of exacerbations, with increasing severity of COPD, impacts the subsequent disease burden of patients with COPD. We conducted a post hoc analysis of the phase III ETHOS study using multi-state modelling to characterise the risk of subsequent severe exacerbations or death based on prior exacerbation history, regardless of treatment [25]. The influence of baseline characteristics on transitions from one event to another, including scenario analysis on outcomes (cumulative exacerbation and death) were explored, and outcomes for different patient profiles were produced.

## Methods

### Study population

The ETHOS study (NCT02465567) evaluated the efficacy and safety of the inhaled corticosteroid (ICS)/long-acting muscarinic antagonist/long-acting β_2_-agonist fixed-dose combination budesonide/glycopyrronium/formoterol fumarate dihydrate metered dose inhaler (BGF MDI; referred to hereafter as BGF) at two budesonide dose levels. Patients with moderate-to-very severe COPD were randomised 1:1:1:1 to BGF 320/18/9.6 μg, BGF 160/18/9.6 μg, glycopyrronium/formoterol fumarate dihydrate MDI (GFF) 18/9.6 μg or budesonide/formoterol fumarate dihydrate MDI (BFF) 320/9.6 μg [25]. All treatments were administered over 52 weeks as two actuations, twice-daily, using an Aerosphere™ inhaler. Triple therapy with BGF 320/18/9.6 μg was found to reduce the risk of moderate or severe exacerbations vs dual therapy with BFF and GFF, and mortality vs GFF [25]. All methods were carried out in accordance with relevant guidelines and regulations, see the ‘Ethics approval and consent to participate’ section.

### Inclusion/exclusion criteria

Inclusion and exclusion criteria have been published previously [25]. Briefly, eligible patients were 40 to 80 years of age and had symptomatic COPD (defined as a score of ≥ 10 on the COPD Assessment Test) with a post-bronchodilator ratio of FEV_1_/forced vital capacity < 0.7, with a post-bronchodilator FEV_1_ of 25% to 65% predicted; and had a documented history of ≥ 1 moderate or severe COPD exacerbations (if their FEV_1_ was < 50% predicted) or ≥ 2 moderate or ≥ 1 severe COPD exacerbations (if their FEV_1_ was ≥ 50% predicted) in the year prior to screening. Patients were excluded from covariate-adjusted analyses if they had any missing baseline covariate data.

### Statistical analysis

This post hoc analysis of the ETHOS study included on-treatment events from the modified intent-to-treat population (mITT; all patients who underwent randomisation, received a study treatment and had post-randomisation data obtained before discontinuation of treatment).

As an initial investigation into the association between exacerbations and mortality, Kaplan–Meier estimates for the proportion of patients dying following exacerbation events were obtained. Survival times were censored at the time of transition to another exacerbation event or time of treatment discontinuation. A hazard ratio (HR) for the risk of death following a severe exacerbation was estimated using a Cox proportional hazards model with an indicator of a severe exacerbation as a time-dependent covariate. Initial analyses made no adjustment for baseline patient characteristics.

For each patient, times of exacerbation events and death were observed exactly or right-censored (i.e., the data points were known to be above a certain value, but it was unknown by how much). Exacerbations were considered distinct events if there were > 7 days between the end of one event and the start of the next. This produced multi-state survival data, where there is a series of event times for each patient, corresponding to times of transition to the next state. At any point in time, a patient occupied one of five well-defined states based upon the events cumulatively experienced while on treatment in the ETHOS study:

- State 1: No exacerbation since study start
- State 2: 1 moderate exacerbation (without severe exacerbation) since study start
- State 3: ≥ 2 moderate exacerbations (without severe exacerbation) since study start
- State 4: ≥ 1 severe exacerbations since study start
- State 5: Death

The last observed state of a patient was either death or censored.

To formally characterise the association between exacerbations and mortality, multi-state modelling based on the Markov assumption (where transition to future states depends only on the current state at time *t* and not previously occupied states) was used [24, 26]. Semi-parametric and fully parametric multi-state models were employed. Each multi-state model estimated transition intensities or transition hazards, representing the instantaneous risk of a patient moving from one state to another in continuous time [24, 27, 28].

**Fig. 1:**
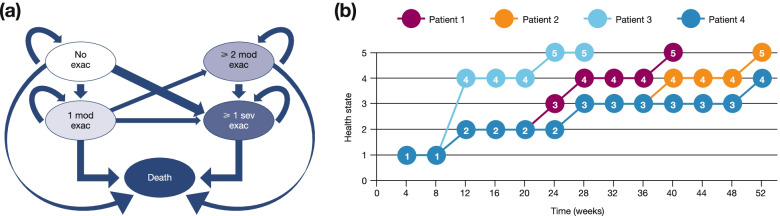
**a** Description of the multi-state model using the Markov assumption; and **b** Paths for four hypothetical patients through the model. All patients were in the ‘no exacerbation’ state at randomisation. The model only allowed transition to a worsened health state. Transitions did not have to be to the next adjacent state; however, transitions directly from no exacerbations to ≥ 2 moderate exacerbations were not permitted. Markov assumption: transition to future states depends only on the current state at time *t* and not previously occupied states. Panel b shows paths for four hypothetical patients: for example, patient 1 (overlapping with patient 2 up to week 20) was in State 1 (no exacerbations) up to week 8, then transitioned to State 2 (1 moderate exacerbation) and stayed there up to week 20. At week 24, the patient transitioned to State 3 (≥ 1 moderate exacerbations) then to State 4 (≥ 1 severe exacerbations) at week 28. The patient remained in State 4 until week 36 and died at week 40. *exac* exacerbation, *mod* moderate, *sev* severe

All patients were in the ‘no exacerbation’ state at randomisation. From this state, the model only allowed a patient to transition to a worsened health state. Transitions did not necessarily have to be to the next adjacent state (i.e. an individual could transition from no exacerbations to death without an exacerbation); however, transitions directly from no exacerbations to ≥ 2 moderate exacerbations were not permitted (Fig. 1). Additional details can be found in the supplementary material.

For the semi-parametric model, a standard Cox proportional hazards regression was fitted to the multi-state data. This specified a different baseline hazard for each transition by including each transition type as separate strata. The baseline hazard was estimated non-parametrically, using the Breslow estimator.

Standard parametric models (exponential, Weibull, Gompertz, log-logistic, log-normal, gamma and generalised gamma) were fitted to the multi-state data and compared using visual assessments and Akaike information criterion (AIC). Times from baseline to transition to next state were modelled, assuming a distinct rate parameter for each transition type. Visual assessments of model fit included comparisons of the Aalen-Johansen estimates [29] of state occupancy probabilities and the Nelson-Aalen estimates [30] of the cumulative hazard (i.e. non-parametric estimates) with estimates based on the fitted parametric models. For each parametric model, state occupancy probabilities were obtained and plotted for various time points up to 5 years, which was beyond the typical duration of an outcome trial in COPD [31–33]. The best fitting parametric model was that which fitted well to the observed data and gave clinically plausible state occupancy probability estimates up to 5 years.

To assess whether risk of death increases as patients accumulate exacerbations, estimated regression coefficients and corresponding standard errors from the best fitting parametric model were used to obtain hazard ratios with 95% confidence intervals (CIs), comparing the risk of death following 1 moderate exacerbation, ≥ 2 moderate exacerbations and ≥ 1 severe exacerbations with the risk of death following no exacerbation. Similarly, hazard ratios were obtained to compare the risk of ≥ 1 severe exacerbations from states of exacerbation with the risk from no exacerbation.

The transition probabilities at 4 weeks from study entry were estimated based on the best fitting parametric multi-state model, without adjustment for covariates. To estimate probabilities of occupying each state at a fixed point in time *t*, we computed a transition probability matrix, where the (*r,s*) entry is the probability of occupying state *s* at time *t*, given the state at time 0 is *r*. Since we used Markov models, this was obtained by solving the Kolmogorov differential equations [34].

To explore the influence of baseline patient characteristics on each of the transition intensities, the multi-state models were adjusted for pre-specified covariates, including treatment, number of exacerbations in previous year, eosinophil count (log-transformed), ICS use at screening, FEV_1_% predicted, sex and age. The pairwise interaction between eosinophil count (log-transformed) and treatment was included in the final covariate-adjusted multi-state model, based on the likelihood ratio test with a significance level < 0.05. The covariate-adjusted models assumed that hazards (transition intensities) were proportional between covariate values/patient groups. Covariate effects were allowed to vary for each transition, with the exception of transitions from exacerbation states (States 2–4) to death, where common effects were assumed due to insufficient data. The transition to death was, for the same reason, assumed to be independent of treatment and sex.

For eight patient profiles defined by FEV_1_ (40% vs 55%), exacerbation at baseline (1 vs 2) and age (55 vs 70 years), transition probabilities at numerous time points within and beyond the study period were calculated based on the best fitting parametric model, adjusted for covariates. These transition probabilities represented the proportion of patients incurring moderate and/or severe exacerbations or dying over up to 5 years.

Since results may be biased if the Markov assumption is violated, as a simple test, we included time spent in State 1 (no exacerbation) as a covariate in the best fitting parametric model for transitions out of exacerbation states (i.e. transitions out of States 2–4). The estimated hazard ratios were all around the null value 1 (Table S1), supporting the Markov assumption, therefore the results presented are based on the fitted Markov models.

Data were analysed using R version 3.6.3 [35]. The “survival” [36] and “mstate” [37] R packages were used for non-parametric and semi-parametric approaches. The “flexsurv” [38] R package was used to fit fully parametric Markov models via maximum likelihood estimation and predict state occupancy probabilities.

## Results

Overall, 8509 patients were included in the mITT population; the summary baseline characteristics are shown in Table 1. Eight patients had missing baseline covariate data and were excluded from the covariate-adjusted analysis. The majority of patients (*n* = 8504) were assigned to State 1 (no exacerbations) at initiation on Day 1. Three patients had moderate exacerbations on Day 1 and two patients had a severe exacerbation on Day 1. These patients were immediately assigned to State 2 (1 moderate exacerbation; three patients) or State 4 (≥ 1 severe exacerbations; two patients). In total, 6081 on-treatment exacerbation events and 134 on-treatment deaths were observed in the final retrieved dataset within 30 days of the last day of treatment. For patients with multiple events, time-to-event from study entry varied, with a median of 48 days (interquartile range, 2–223 days).

**Table 1:** Summary of study population characteristics at baseline (mITT population)

	**All patients** **(*N* = 8509)**
Age, years, mean (SD)	64.7 (7.6)
Male, *n* (%)	5081 (59.7)
Treatment arm, *n* (%)	
BGF 320/18/9.6 µg	2137 (25.1)
BGF 160/18/9.6 µg	2121 (24.9)
GFF 18/9.6 µg	2120 (24.9)
BFF 320/9.6 µg	2131 (25.0)
ICS use, *n* (%)	6846 (80.5)
Number of moderate or severe exacerbations in last year, median (range)	2 (0–12)
1, *n* (%)	3699 (43.5)
≥ 2, *n* (%)	4810 (56.5)
Blood eosinophil count, mean × 10^9^/L (SD)	196.4 (132.8)
Post-bronchodilator FEV_1_% predicted, mean % (SD)	43.4 (10.3)

*BFF* Budesonide/formoterol, *BGF* Budesonide/glycopyrronium/formoterol, *FEV*_*1*_ Forced expiratory volume in 1 s, *GFF* Glycopyrronium/formoterol, *ICS* Inhaled corticosteroid, *mITT* Modified intent-to-treat, *SD* Standard deviation

Of the 134 on-treatment deaths, 42 (31.3%) occurred after patients had a severe exacerbation (Table S2), with one further death occurring on the same day as a severe exacerbation; 76 (56.7%) occurred after ≥ 1 moderate or severe exacerbations. Of note, 43% of patients died without having any prior exacerbation.

**Fig. 2:**
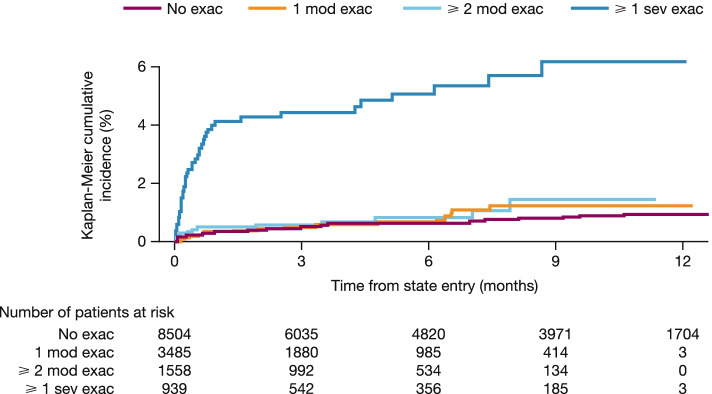
Kaplan–Meier curves for time to death (cumulative incidence) from time of entry to four progressive exacerbation states arising post-randomisation (mITT population). Since time is measured from state entry, and the majority of patients are in the state of no exacerbation at study entry, the number at risk is much higher at 12 months for the no exacerbation state compared with the other exacerbation states. *exac* exacerbation, *mITT* modified intent-to-treat, *mod* moderate, *sev* severe

Probabilities of death (cumulative incidence) were higher following a severe exacerbation, particularly during the month immediately following the event (acute effect) and continuing beyond the first month (chronic effect; as shown in Fig. 2), with an estimated higher risk of death (HR 8.3 [95% CI, 5.59–12.3]) following a severe exacerbation vs no exacerbations. According to visual comparisons of estimates of state occupancy probabilities (Fig. S1) and estimates of cumulative hazards for each transition type (Fig. S2), the exponential model was a poor fit to the observed data compared with alternative parametric models. Although the Gompertz model was the best fit to the observed data based on AIC (Table S3), probability estimates beyond the trial period were much lower in the longer term than would be expected from long-term studies in COPD (Table S4, Fig. S3) [31–33]. The generalised gamma, Weibull and gamma models showed a good fit to the observed data, gave similarly plausible estimates of state occupancy up to 5 years, similar visual estimates and AIC, and, thus, were preferred over the Gompertz model. We present results based on the Weibull model so as to enable reporting of hazard ratios.

The unadjusted Weibull model estimated that death following a severe exacerbation was 11.4 times (95% CI, 7.7–17.0), 6.4 times (95% CI, 3.8–10.8) and 5.4 times (95% CI, 2.9–10.3) more likely than death following no exacerbations, 1 moderate exacerbation or ≥ 2 moderate exacerbations, respectively (Table 2). One moderate exacerbation made death 1.8 times (95% CI, 1.1–2.9) more likely than death following no exacerbations, with ≥ 2 moderate exacerbations making death 2.1 times (95% CI, 1.1–3.9) more likely than death following no exacerbations.

**Table 2:** Hazard ratios comparing risks of death and ≥ 1 severe exacerbation among the different states based on the best fitting Weibull multi-state Markov model without adjustment for covariates (mITT population)

Transition to death from	vs transition to death from	HR	SE	95% CI
State 2 (1 moderate exacerbation)	State 1 (No exacerbation)	1.8	0.3	(1.1–2.9)
State 3 (≥ 2 moderate exacerbations)	State 1 (No exacerbation)	2.1	0.3	(1.1–3.9)
State 3 (≥ 2 moderate exacerbations)	State 2 (1 moderate exacerbation)	1.2	0.4	(0.6–2.4)
State 4 (≥ 1 severe exacerbations)	State 1 (No exacerbation)	11.4	0.2	(7.7–17.0)
State 4 (≥ 1 severe exacerbations)	State 2 (1 moderate exacerbation)	6.4	0.3	(3.8–10.8)
State 4 (≥ 1 severe exacerbations)	State 3 (≥ 2 moderate exacerbations)	5.4	0.3	(2.9–10.3)
**Transition to ≥ 1 severe exacerbation from**	**vs transition to ≥ 1 severe exacerbation from**			
State 2 (1 moderate exacerbation)	State 1 (No exacerbation)	1.4	0.1	(1.2–1.6)
State 3 (≥ 2 moderate exacerbations)	State 1 (No exacerbation)	1.9	0.1	(1.6–2.3)
State 3 (≥ 2 moderate exacerbations)	State 2 (1 moderate exacerbation)	1.4	0.1	(1.1–1.7)

*HR* Hazard ratio, *CI* Confidence interval, *mITT* Modified intent-to-treat, *SE* Standard error for the log hazard ratio

Markov assumption: transition to future states depends only on the current state at time *t* and not previously occupied states

Using the Weibull multi-state Markov model without covariate adjustment, probabilities of having a severe exacerbation in four weeks’ time were 147 per 10,000 patients with no exacerbations, and higher at 205 and 274 per 10,000 patients for those with 1 moderate and ≥ 2 moderate exacerbations, respectively (Table 3). Probabilities of death were 15, 25 and 29 per 10,000 patients over 4 weeks, following no exacerbations, 1 moderate exacerbation and ≥ 2 moderate exacerbations, respectively. The probability of death was higher at 149 per 10,000 patients for those that had a severe exacerbation.

**Table 3:** Estimated transition probabilities from baseline to 4 weeks based on the Weibull multi-state model using the Markov assumption^a^ and fitted without covariates, reported per 10,000 patients (mITT population)

From exacerbation state	To exacerbation state				
	**State 1** **(No exacerbation)**	**State 2** **(1 moderate exacerbation)**	**State 3** **(≥ 2 moderate exacerbations)**	**State 4** **(≥ 1 severe exacerbations)**	**State 5** **(Death)**
State 1 (No exacerbation)	9085 (9027–9136)	693 (655–734)	61 (53–70)	147 (134–161)	15 (12–18)
State 2 (1 moderate exacerbation)	0 (0–0)	8256 (8115–8383)	1514 (1396–1632)	205 (176–238)	25 (18–37)
State 3 (≥ 2 moderate exacerbations)	0 (0–0)	0 (0–0)	9696 (9636–9747)	274 (227–330)	29 (17–50)
State 4 (≥ 1 severe exacerbations)	0 (0–0)	0 (0–0)	0 (0–0)	9851 (9795–9894)	149 (106–205)
State 5 (Death)	0 (0–0)	0 (0–0)	0 (0–0)	0 (0–0)	10,000 (10,000–10,000)

95% CIs are reported and were obtained by simulating 1000 samples from the asymptotic normal distribution of the maximum likelihood estimates [38]

*CI* Confidence interval, *mITT* Modified intent-to-treat

^a^Markov assumption is that transition to future states depends only on the current state at time *t* and not previously occupied states

The multi-state model estimated that having a severe exacerbation following ≥ 2 moderate exacerbations was 1.9 times (95% CI, 1.6–2.3) more likely than following no exacerbations, and 1.4 times (95% CI, 1.1–1.7) more likely than following 1 moderate exacerbation (Table 2). One moderate exacerbation increased the likelihood of a severe exacerbation by 1.4 times (95% CI, 1.2–1.6) compared with no exacerbation.

**Fig. 3:**
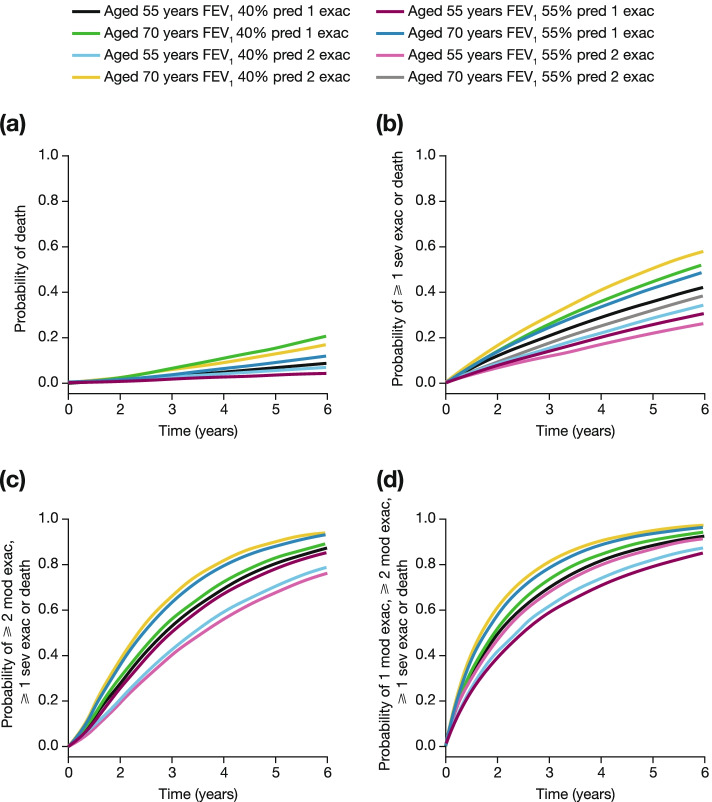
Estimated probabilities of occupying states of **a** death, **b** ≥ 1 severe exacerbations or death, **c** ≥ 2 moderate exacerbations, ≥ 1 severe exacerbations or death, and **d** 1 moderate exacerbation, ≥ 2 moderate exacerbations, ≥ 1 severe exacerbations or death, estimated for eight patient profiles, based on patient age, FEV_1_% predicted and exacerbation history, given the patients occupied the state of no exacerbation at study entry (time 0). *exac* exacerbation, *FEV*_*1*_ forced expiratory volume in 1 s, *mITT* modified intent-to-treat, *mod* moderate, *pred* predicted, *sev* severe

Figure 3 shows the estimated probabilities of occupying different possible states for eight patient profiles, defined by patient age (55 vs 70 years), FEV_1_% predicted (55 vs 40%) and exacerbation history (1 vs 2 exacerbations in the prior year). Probabilities of death were noticeably higher for 70-year-old patients with FEV_1_ 40% predicted (Fig. 3a) although based upon limited data on deaths. The probability of death or ≥ 1 severe exacerbations showed a clear, distinct ranking between patients (Fig. 3b). The number of exacerbations in the previous year affected the transition intensities in the Markov model (Table S5). For patients with the same FEV_1_% predicted normal and age, each additional exacerbation was associated with increased risk of incurring a severe exacerbation or death; the estimated probability at 5 years for a 70-year-old with FEV_1_ 40% predicted was 53% for patients with one prior exacerbation and 59% for patients with two prior exacerbations. When considering probabilities of occupying one of three or four disease states (Fig. 3c and 3d), older patients aged 70 years with FEV_1_ 40% predicted and 2 exacerbations in the prior year were at increased risk.

## Discussion

Using a multi-state model under the Markov assumption, there was an estimated 11-fold increased risk of death following a severe exacerbation compared with no exacerbations, and an approximately 2-fold increased risk of death following one exacerbation compared with no exacerbations. Even patients with one moderate exacerbation were found to have a higher risk of severe exacerbation compared with patients who had no moderate exacerbations; the risk was even higher in patients with a second moderate exacerbation. In particular, patients who incurred one or more severe exacerbations or had increasing numbers of moderate exacerbations were at a greater risk of all-cause mortality compared with all the other health states. These findings are in line with a previous study by Rothnie et al. which used Cox modelling to predict risk of future severe exacerbations and mortality with increasing numbers of moderate exacerbations [23]. Together, these studies illustrate the importance of preventing exacerbations of any severity, particularly severe exacerbations, given the increased risk of morbidity and all-cause mortality subsequent to these events [16–21]. Furthermore, although exacerbations (moderate or severe) were shown to increase the risk of hospitalisation or death, it is also notable that almost half of patients who died during the study did not have a prior exacerbation.

Multi-state Markov models are typically used to evaluate progression between COPD states defined by Global Initiative for Chronic Obstructive Lung Disease airflow limitation severities rather than exacerbations risk. In addition, they are often utilised in health economics and policy models to project epidemiological data and outcomes in terms of mortality, costs and life benefits, among others. For this analysis, using a multi-state model was beneficial as it allowed estimation of the risk of events (exacerbations and/or deaths) for different patient profiles. The parametric models developed were useful to assess the risk of exacerbations and all-cause mortality beyond the time horizon of observation and may inform clinical decision making and assessment of the burden of COPD at an individual level.

There were a number of limitations to this study. The ETHOS study recruited a population of patients with moderate-to-very severe COPD who had a history of exacerbations, therefore, it is not possible to predict risk profiles for patients without an exacerbation history. While Markov models are very useful in describing disease processes as discrete states, some transitions in this analysis had relatively sparse data, meaning that some Markov models were fitted based on assumptions. Specifically, it was assumed that there was no effect of treatment and sex on transitions to death, and common effects of the remaining covariates on transitions from exacerbation states to death. In fact, a 49% reduction in risk of mortality was observed in the ETHOS study for BGF 320/18/9.6 µg relative to GFF (HR 0.51 [95% CI, 0.33–0.80]) [39]. Although unable to account for such treatment effects on death, the model was able to represent the increased burden from prior events, which was the main aim of the work.

Another limitation is that although overall the chosen fully parametric model was a good fit to the observed data based on agreement between non-parametric and parametric estimates of state occupancy probabilities, there were some discrepancies between non-parametric and parametric estimates of cumulative hazard for a few transition types, where data are sparse. Future work could explore more advanced modelling approaches such as for the parametric distribution to differ according to the type of transition. Another extension to this work could be to predict outcomes over a longer-term duration of a COPD patient’s survival, using multi-state modelling techniques in combination with elicited expert opinion or external data [40].

## Conclusion

Multi-state modelling using data from the ETHOS study quantifies the cumulative burden of exacerbations in patients with COPD and demonstrates the extent to which the risk of all-cause mortality increases following a severe exacerbation and the extent to which the risk of a severe exacerbation increases as patients accumulate moderate exacerbations. These exploratory analyses showed that there was an acute (i.e. first month after a severe exacerbation) and chronic (i.e. following the first month after a severe exacerbation) effect of severe exacerbations on mortality risk. In addition, the mortality risk after a moderate exacerbation was higher than before a moderate exacerbation. The impact of just one moderate exacerbation on mortality risk in patients with COPD highlights that clinical management with novel pharmacotherapies should be optimised to prevent even moderate exacerbations. Estimated transition probabilities may provide greater granularity in quantifying patient burden and subsequent costs.

## Supplementary Information

**Additional file 1.**

## Data Availability

Data underlying the findings described in this manuscript may be obtained through Vivli’s web-based data request platform (https://www.vivli.org) in accordance with AstraZeneca’s data sharing policy described at https://www.astrazenecagrouptrials.pharmacm.com/ST/Submission/Disclosure.

## Abbreviations

AIC: Akaike information criterion
BFF: Budesonide/formoterol
BGF: Budesonide/glycopyrronium/formoterol
CI: Confidence interval
COPD: Chronic obstructive pulmonary disease
FEV_1_: Forced expiratory volume in 1 s
GFF: Glycopyrronium/formoterol
HR: Hazard ratio
ICS: Inhaled corticosteroid
MDI: Metered dose inhaler
mITT: Modified intent-to-treat
SD: Standard deviation
SE: Standard error

## References

[1] Halpin DMG, Celli BR, Criner GJ, Frith P, López Varela MV, Salvi S, Vogelmeier CF, Chen R, Mortimer K, Montes de Oca M (2019). The GOLD Summit on chronic obstructive pulmonary disease in low- and middle-income countries. Int J Tuberc Lung Dis.

[2] GBD 2017 Disease and Injury Incidence and Prevalence Collaborators (2018). Global, regional, and national incidence, prevalence, and years lived with disability for 354 diseases and injuries for 195 countries and territories, 1990–2017: a systematic analysis for the Global Burden of Disease Study 2017. Lancet.

[3] Haroon S, Adab P, Riley RD, Fitzmaurice D, Jordan RE (2017). Predicting risk of undiagnosed COPD: development and validation of the TargetCOPD score. Eur Respir J.

[4] Global Initiative for Chronic Obstructive Lung Disease. Global strategy for the diagnosis, management, and prevention of chronic obstructive pulmonary disease. 2021 report. 2021. https://goldcopd.org/archived-reports/. Accessed 5 Apr 2021.

[5] Celli BR, MacNee W (2004). ATS/ERS Task Force. Standards for the diagnosis and treatment of patients with COPD: a summary of the ATS/ERS position paper. Eur Respir J.

[6] Press VG, Konetzka RT, White SR (2018). Insights about the economic impact of chronic obstructive pulmonary disease readmissions post implementation of the hospital readmission reduction program. Curr Opin Pulm Med.

[7] Burge S, Wedzicha JA (2003). COPD exacerbations: definitions and classifications. Eur Respir J.

[8] Andersson F, Borg S, Jansson SA, Jonsson AC, Ericsson A, Prütz C, Rönmark E, Lundbäck B (2002). The costs of exacerbations in chronic obstructive pulmonary disease (COPD). Respir Med.

[9] Hartl S, Lopez-Campos JL, Pozo-Rodriguez F, Castro-Acosta A, Studnicka M, Kaiser B, Roberts CM (2016). Risk of death and readmission of hospital-admitted COPD exacerbations: European COPD Audit. Eur Respir J.

[10] Janson C, Nwaru BI, Wiklund F, Telg G, Ekström M (2020). Management and risk of mortality in patients hospitalised due to a first severe COPD exacerbation. Int J Chron Obstruct Pulmon Dis.

[11] Patel JG, Coutinho AD, Lunacsek OE, Dalal AA (2018). COPD affects worker productivity and health care costs. Int J Chron Obstruct Pulmon Dis.

[12] Miravitlles M, Peña-Longobardo LM, Oliva-Moreno J, Hidalgo-Vega Á. Caregivers’ burden in patients with COPD. Int J Chron Obstruct Pulmon Dis. 2015;10:347–56. 10.2147/COPD.S76091 PMC4334315 25709429

[13] Nakken N, Janssen DJA, van den Bogaart EHA, Wouters EFM, Franssen FME, Vercoulen JH, Spruit MA (2015). Informal caregivers of patients with COPD: home sweet home?. Eur Respir Rev.

[14] Müllerová H, Shukla A, Hawkins A, Quint J (2014). Risk factors for acute exacerbations of COPD in a primary care population: a retrospective observational cohort study. BMJ Open.

[15] Hurst JR, Vestbo J, Anzueto A, Locantore N, Müllerová H, Tal-Singer R, Miller B, Lomas DA, Agusti A, Macnee W (2010). Susceptibility to exacerbation in chronic obstructive pulmonary disease. N Engl J Med.

[16] Haughney J, Lee AJ, Nath M, Müllerová H, Holmgren U, Nigris E, Ding B. The long-term clinical impact of COPD exacerbations: a 3-year observational study (SHERLOCK). Ther Adv Respir Dis. 2022;16:17534666211070140. 10.1177/17534666211070139 PMC8848076 35156488

[17] Suissa S, Dell’Aniello S, Ernst P. Long-term natural history of chronic obstructive pulmonary disease: severe exacerbations and mortality. Thorax. 2012;67(11):957–63. 10.1136/thoraxjnl-2011-201518 PMC3505864 22684094

[18] Kerkhof M, Voorham J, Dorinsky P, Cabrera C, Darken P, Kocks JWH, Sadatsafavi M, Sin DD, Carter V, Price DB (2020). The long-term burden of COPD exacerbations during maintenance therapy and lung function decline. Int J Chron Obstruct Pulmon Dis.

[19] Rothnie KJ, Connell O, Müllerová H, Smeeth L, Pearce N, Douglas I, Quint JK (2018). Myocardial infarction and ischemic stroke after exacerbations of chronic obstructive pulmonary disease. Ann Am Thorac Soc.

[20] Hurst JR, Skolnik N, Hansen GJ, Anzueto A, Donaldson GC, Dransfield MT, Varghese P (2020). Understanding the impact of chronic obstructive pulmonary disease exacerbations on patient health and quality of life. Eur J Intern Med.

[21] Barnes N, Calverley PMA, Kaplan A, Rabe KF (2013). Chronic obstructive pulmonary disease and exacerbations: patient insights from the global Hidden Depths of COPD survey. BMC Pulm Med.

[22] Andersen PK, Gill RD. Cox’s regression model for counting processes: a large sample study. Ann Statist. 1982;10(4):1100–20.

[23] Rothnie KJ, Müllerová H, Smeeth L, Quint JK (2018). Natural history of chronic obstructive pulmonary disease exacerbations in a general practice-based population with chronic obstructive pulmonary disease. Am J Respir Crit Care Med.

[24] Meira-Machado L, de Uña-Alvarez J, Cadarso-Suárez C, Andersen PK (2009). Multi-state models for the analysis of time-to-event data. Stat Methods Med Res.

[25] Rabe KF, Martinez FJ, Ferguson GT, Wang C, Singh D, Wedzicha JA, Trivedi R, St Rose E, Ballal S, McLaren J (2020). Triple inhaled therapy at two glucocorticoid doses in moderate-to-very-severe COPD. N Engl J Med.

[26] Saint-Pierre P, Combescure C, Daurès JP, Godard P (2003). The analysis of asthma control under a Markov assumption with use of covariates. Stat Med.

[27] Jackson CH (2011). Multi-state models for panel data: the msm package for R. J Stat Softw.

[28] Andersen PK, Keiding N (2002). Multi-state models for event history analysis. Stat Methods Med Res.

[29] Aalen OO, Johansen S (1978). An empirical transition matrix for non-homogeneous Markov chains based on censored observations. Scand J Statist.

[30] Nelson W (1969). Hazard plotting for incomplete failure data. J Qual Technol.

[31] Vestbo J, Anderson JA, Brook RD, Calverley PMA, Celli BR, Crim C, Martinez F, Yates J, Newby DE, SUMMIT Investigators (2016). Fluticasone furoate and vilanterol and survival in chronic obstructive pulmonary disease with heightened cardiovascular risk (SUMMIT): a double-blind randomised controlled trial. Lancet.

[32] Tashkin DP, Celli B, Senn S, Burkhart D, Kesten S, Menjoge S, Decramer M (2008). A 4-year trial of tiotropium in chronic obstructive pulmonary disease. N Engl J Med.

[33] Calverley PMA, Anderson JA, Celli B, Ferguson GT, Jenkins C, Jones PW, Yates JC, Vestbo J (2007). TORCH Investigators. Salmeterol and fluticasone propionate and survival in chronic obstructive pulmonary disease. N Engl J Med.

[34] Cox DR, Miller HD (1965). The Theory of Stochastic Processes.

[35] R Core Team. R: a language and environment for statistical computing. 2018. https://www.R-project.org/. Accessed 9 Oct 2021.

[36] Therneau TM, Grambsch PM (2000). Modeling Survival Data: Extending the Cox Model.

[37] Putter H, Fiocco M, Geskus RB (2007). Tutorial in biostatistics: competing risks and multi-state models. Stat Med.

[38] Jackson CH (2016). flexsurv: a platform for parametric survival modeling in R. J Stat Softw.

[39] Martinez FJ, Rabe KF, Ferguson GT, Wedzicha JA, Singh D, Wang C, Rossman K, St Rose E, Trivedi R, Ballal S (2021). Reduced all-cause mortality in the ETHOS trial of budesonide/glycopyrrolate/formoterol for chronic obstructive pulmonary disease. a randomized, double-blind, multicenter, parallel-group study. Am J Respir Crit Care Med.

[40] Jackson C, Stevens J, Ren S, Latimer N, Bojke L, Manca A, Sharples L (2017). Extrapolating survival from randomized trials using external data: a review of methods. Med Decis Making.

[41] Battisti WP, Wager E, Baltzer L, Bridges D, Cairns A, Carswell CI, Citrome L, Gurr JA, Mooney LA, Moore BJ (2015). Good publication practice for communicating company-sponsored medical research: GPP3. Ann Intern Med.

