# Supplementary material for: Relationship between risk, cumulative burden of exacerbations and mortality in patients with COPD: modelling analysis using data from the ETHOS study
Source: BMC Med Res Methodol. 2022 May 25;22:150. doi: 10.1186/s12874-022-01616-7 (PMC9134588; doi:10.1186/s12874-022-01616-7)
Supplement: Supplementary file 1 — Additional file 1. [file 12874_2022_1616_MOESM1_ESM.docx]

# Supplementary Information

**Table S1** Associations between time spent in State 1 (no exacerbation) and state transitions

| **State transition** | **HR (95% CI)** |
| --- | --- |
| State 1 – State 2 (None – 1 moderate exacerbation) | 1.000 (1.000–1.000) |
| State 1 – State 4 (None – ≥ 1 severe exacerbations) | 1.000 (1.000–1.000) |
| State 1 – State 5 (None – death) | 1.000 (1.000–1.000) |
| State 2 – State 3 (1 moderate exacerbation – ≥ 2 moderate exacerbations) | 0.999 (0.998–1.000) |
| State 2 – State 4 (1 moderate exacerbation – ≥ 1 severe exacerbations) | 1.000 (0.998–1.001) |
| State 2 – State 5 (1 moderate exacerbation – death) | 1.002 (0.997–1.007) |
| State 3 – State 4 (≥ 2 moderate exacerbations – ≥ 1 severe exacerbations) | 1.001 (0.998–1.004) |
| State 3 – State 5 (≥ 2 moderate exacerbations – death) | 1.006 (0.998–1.014) |
| State 4 – State 5 (≥ 1 severe exacerbation – death) | 1.004 (1.000–1.007) |

CI confidence interval, HR hazard ratio

**Table S2** Number of observed on-treatment exacerbation events/deaths by previous exacerbation state (mITT population)

| **Previous exacerbation state** | **State 2** **(1 moderate exacerbation)**  ***N* = 3529** | **State 3** **(≥ 2 moderate exacerbations)** ***N* = 1593** | **State 4**  **(≥ 1 severe exacerbations)** ***N* = 959** | **State 5**  **(Death)** ***N* = 134** |
| --- | --- | --- | --- | --- |
| State 1, *n* (%) (None) | 3529 (100) | 0 | 646 (67.4) | 58 (43.3) |
| State 2, *n* (%) (1 moderate exacerbation) | 0 | 1593 (100) | 191 (19.9) | 22 (16.4) |
| State 3, *n* (%) (≥ 2 moderate exacerbations) | 0 | 0 | 122 (12.7) | 12 (9.0) |
| State 4, *n* (%) (≥ 1 severe exacerbations) | 0 | 0 | 0 | 42 (31.3) |

*mITT* modified intent-to-treat, *n* number of events

**Table S3** Comparison of unadjusted parametric multi-state models based on AIC

| **Distribution** | **AIC** |
| --- | --- |
| Gompertz  Generalised gamma  Weibull  Log-logistic  Gamma  Log-normal  Exponential | 92,113  92,164  92,213  92,225  92,244  92,329  92,428 |

AIC Akaike information criterion

**Table S4** Estimated percentages of patients occupying states of death; ≥ 1 severe exacerbations or death; ≥ 2 moderate exacerbations, ≥ 1 severe exacerbations or death; and 1 moderate exacerbation, ≥ 2 moderate exacerbations, ≥ 1 severe exacerbations or death, based on the fitted unadjusted models, given that patients occupied the state of no exacerbation at study entry (time 0)

| **State(s)** | **Time** | **Non-parametric (95% CI)** | **Exponential** | **Weibull** | **Log-normal** | **Log-logistic** | **Gompertz** | **Gamma** | **Generalised gamma** |
| --- | --- | --- | --- | --- | --- | --- | --- | --- | --- |
| Death | 3 months | 0.4 (0.3–0.5) | 0.3 | 0.4 | 0.2 | 0.3 | 0.5 | 0.5 | 0.3 |
|  | 6 months | 0.8 (0.6–1.1) | 0.8 | 0.9 | 0.7 | 0.8 | 1.0 | 0.9 | 0.8 |
|  | 9 months | 1.4 (1.1–1.6) | 1.3 | 1.4 | 1.3 | 1.3 | 1.5 | 1.4 | 1.3 |
|  | 1 year | 1.9 (1.5–2.2) | 1.9 | 2.0 | 2.1 | 1.9 | 2.0 | 2.0 | 2.0 |
|  | 2 years | – | 5.1 | 4.5 | 5.5 | 4.8 | 3.4 | 4.5 | 4.8 |
|  | 3 years | – | 9.4 | 7.5 | 9 | 8.2 | 10.9 | 7.5 | 8 |
|  | 4 years | – | 14.2 | 10.7 | 12.3 | 11.6 | 4.4 | 10.9 | 11.2 |
|  | 5 years | – | 19.5 | 14.1 | 15.5 | 15 | 4.5 | 14.4 | 14.5 |
| ≥ 1 severe exacerbations or death | 3 months | 4.3 (3.9–4.9) | 3.4 | 4.3 | 3.3 | 3.7 | 4.4 | 4.4 | 3.7 |
|  | 6 months | 8.0 (7.2–8.8) | 6.9 | 7.7 | 7.4 | 7.3 | 8.1 | 7.8 | 7.4 |
|  | 9 months | 11.3 (10.3–12.2) | 10.4 | 10.9 | 11.3 | 10.8 | 11.3 | 10.9 | 10.9 |
|  | 1 year | 13.9 (12.9–14.9) | 14.0 | 14.0 | 15.0 | 14.1 | 13.9 | 14.0 | 14.1 |
|  | 2 years | – | 28 | 25.3 | 26.7 | 26 | 20.5 | 25.4 | 25.7 |
|  | 3 years | – | 40.9 | 35.2 | 35.6 | 35.6 | 23.3 | 35.8 | 35.2 |
|  | 4 years | – | 52 | 43.9 | 42.6 | 43.3 | 24.6 | 44.9 | 43.1 |
|  | 5 years | – | 61.3 | 51.3 | 48.2 | 49.6 | 25.1 | 52.9 | 50 |
| ≥ 2 moderate exacerbations,  ≥ 1 severe exacerbations or death | 3 months | 7.8 (6.8–8.7) | 5.7 | 7.9 | 8.2 | 7.8 | 8.1 | 7.7 | 7.7 |
|  | 6 months | 18.0 (16.6–19.5) | 14.2 | 16.7 | 17.7 | 16.9 | 17.8 | 16.4 | 17.0 |
|  | 9 months | 26.5 (24.7–28.3) | 24.0 | 25.4 | 25.9 | 25.1 | 26.3 | 25.2 | 25.6 |
|  | 1 year | 33.5 (31.5–35.5) | 33.8 | 33.6 | 32.7 | 32.1 | 33.2 | 33.7 | 33.2 |
|  | 2 years | – | 65.7 | 59.2 | 51.8 | 51.8 | 48.7 | 60.9 | 55.9 |
|  | 3 years | – | 83.7 | 75 | 63.2 | 63.7 | 54.7 | 77.6 | 69.8 |
|  | 4 years | – | 92.5 | 84.6 | 70.9 | 71.5 | 57.1 | 87.3 | 78.6 |
|  | 5 years | – | 96.6 | 90.4 | 76.3 | 76.9 | 58.1 | 92.8 | 84.5 |
| 1 moderate exacerbation, ≥ 2 moderate exacerbations,  ≥ 1 severe exacerbations or death | 3 months | 24.0 (22.2–25.8) | 18.4 | 22.3 | 22.9 | 22.0 | 22.6 | 22.0 | 22.2 |
|  | 6 months | 37.5 (35.2–39.9) | 33.6 | 35.9 | 37.1 | 36.5 | 37.2 | 35.6 | 36.3 |
|  | 9 months | 47.1 (44.5–50.0) | 45.8 | 46.2 | 46.8 | 47.0 | 47.0 | 46.0 | 46.5 |
|  | 1 year | 53.2 (50.3–56.1) | 55.9 | 54.3 | 54.0 | 54.8 | 53.8 | 54.4 | 54.3 |
|  | 2 years | – | 80.5 | 74.9 | 70.7 | 72.8 | 67.0 | 75.9 | 73.3 |
|  | 3 years | – | 91.4 | 85.4 | 79.3 | 81.5 | 71.5 | 86.8 | 82.9 |
|  | 4 years | – | 96.2 | 91.2 | 84.4 | 86.5 | 73.3 | 92.7 | 88.4 |
|  | 5 years | – | 98.3 | 94.6 | 87.8 | 89.7 | 74.0 | 95.9 | 91.9 |

*CI* confidence interval

**Table S5** Associations between the number of exacerbations in the previous year and state transitions under the covariate-adjusted Weibull model

| **State transition** | **HR (95% CI)** |
| --- | --- |
| State 1 – State 2 (None – 1 moderate exacerbation) | 1.29 (1.24–1.33) |
| State 1 – State 4 (None – ≥ 1 severe exacerbations) | 1.13 (1.03–1.24) |
| State 1 – State 5 (None – death) | 0.97 (0.68–1.39) |
| State 2 – State 3 (1 moderate exacerbation – ≥ 2 moderate exacerbation**s**) | 1.18 (1.13–1.24) |
| State 2 – State 4 (1 moderate exacerbation – ≥ 1 severe exacerbations) | 1.16 (1.02–1.32) |
| State 2 – State 5 (1 moderate exacerbation – death) | 0.85 (0.64–1.11) |
| State 3 – State 4 (≥ 2 moderate exacerbations – ≥ 1 severe exacerbations) | 1.26 (1.13–1.42) |
| State 3 – State 5 (≥ 2 moderate exacerbations – death) | 0.85 (0.64–1.11) |
| State 4 – State 5 (≥ 1 severe exacerbations – death) | 0.85 (0.64–1.11) |

CI confidence interval, HR hazard ratio

**Fig. S1** Estimated probabilities of occupying each state over time


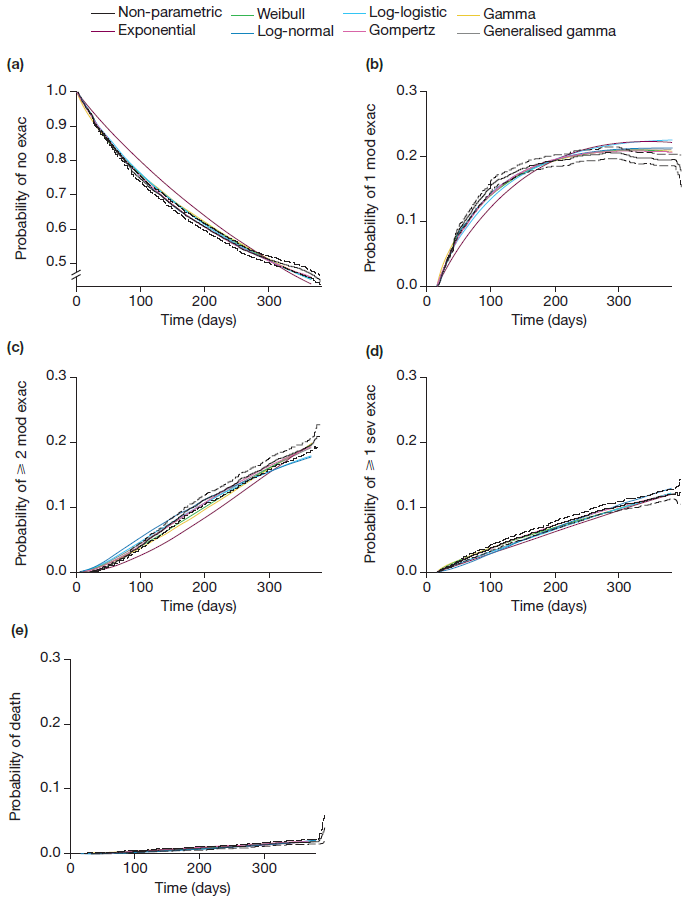


Individuals were in State 1 (no exacerbation) at study entry (time 0), under non-parametric and parametric models. Dashed lines correspond to 95% CIs of non-parametric estimates
*CI* confidence interval, *exac* exacerbation, *mod* moderate, *sev* severe

**Fig. S2** Cumulative transition-specific hazards under non-parametric and parametric models


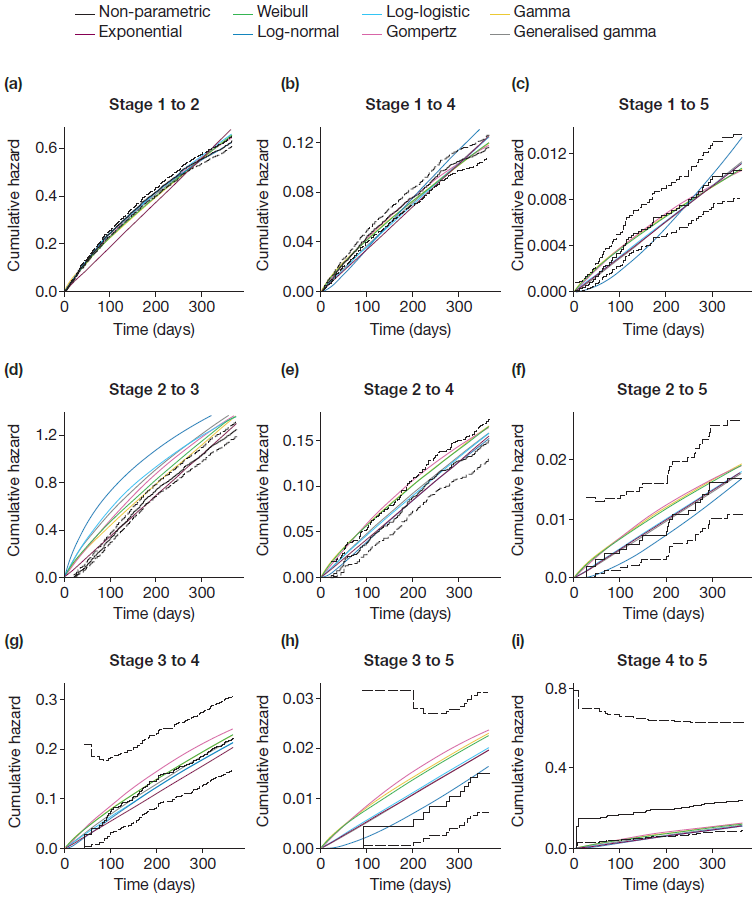


Dashed lines correspond to 95% CIs of non-parametric estimates
*CI* confidence interval

**Fig. S3** Estimated cumulative probabilities of state occupancy over time up to 5 years


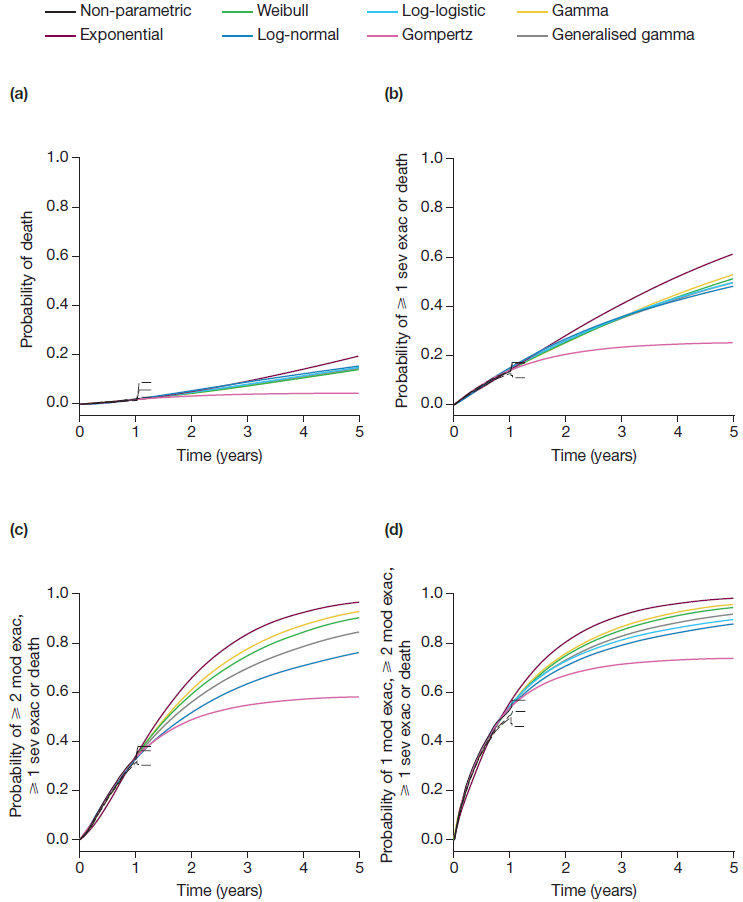


Individuals were in State 1 (no exacerbation) at study entry (time 0), under non-parametric and parametric models
*exac* exacerbation, *mod* moderate, *sev* severe

| **Independent Ethics Committees/Institutional Review Boards consulted** | | |
| --- | --- | --- |
| **Centre no.** | **Name and address of Independent Ethics Committee/Institutional Review Board** | **Chairman of Independent Ethics Committee/ Institutional Review Board** |
| **Argentina** |  |  |
| 005621, 005623, 005626, 005631, 005634, 005635, 005637, 005711, 005785, 005839, 005851, 005875, 005876, 005877 | Comité de Ética en Investigacion INAER Arenales 3146 1º A (C1425BEN), Ciudad Autónoma de Buenos Aires, Argentina | Dr. Gustavo H. Cerezo |
| 005622 | CCIS and Local EC Comité de Ética de CER Investigaciones Clinicas (CECIC) Pdte. Perón Nº 489 2ºC (CP1878) Quilmes Oeste, Bs. As. Argentina | Del Percio Damián |
| 005624 | Comité de ética “Dr. Claude Bernard” Ave Pulmo, Alvear 3345, Mar del Plata, Buenos Aires, Argentina | Daniel Sarcuno |
| 005625, 005632 | Comité Independiente de Ética para ensayos en farmacología clínica CIEFC Av Cabildo1536 piso 5º B CABA (C1426ABP), Buenos Aires, Argentina | Dr. Diego José Zárraga |
| 005628 | CCIS and Local EC: Comité de Bioética Cimel Tucumán 1314 Lanús Este, Buenos Aires, Argentina | Dr. Fabian Augusto Gamallo |
| 005629, 005717 | Comité Independiente de Ética de Investigación en Salud Prof. Dr. Marcelino Rusculleda Av. Colon 2057 Córdoba (X5003DCE), Córdoba, Argentina | Dr. Carlos J. Alfonso |
| 005622, 005627, 005716 | CCIS and Local EC Comité de Ética de CER Investigaciones Clinicas (CECIC) Pdte. Perón Nº 489 2ºC (CP1878), Quilmes Oeste, Bs. As. Argentina | Del Percio Damián |
| 005630 | Comité Independiente de Ética Iniciativa y Reflexión Bioética Rosario Rioja 2926, Rosario, Santa Fé (S2002OJN), Argentina | Dr. Pablo Valarino |
| 005633 | CCIS and Local EC: Comité de Ética en Investigación Instituto de Investigaciones Clínicas Av. Colón 3364, B7600FZN, Mar del Plata, Argentina | Prof. Dr. Jesus Vazquez |
| 005636 | CCIS and Local EC: CEI CIM-MDP Av. Colón 3083. Piso 5. CP 7600. Mar del Plata, Buenos Aires, Argentina | Dr. Martin Donadio |
| 005638 | CCIS and Local EC: Comité de Ética y Docencia de Diagnóstico Maipú (CEDIMA) Av. Pte. Perón 999, San Fernando, Buenos Aires, Argentina | Diego Javier Gremes |
| 005639 | CCIS and Local EC: Comité de Ética en Investigación Instituto Ave Pulmo, Alvear 3345, Mar del Plata, Buenos Aires, Argentina | Dr. Carlos Maria de la Vega |
| 005788 | Comité Independiente de Ética Médica del Noroeste Argentino Las Piedras 496, 4°piso, San Miguel de Tucumán, Tucumán, Argentina | Dr. Oscar E. Gallardo |
| 005786, 005789 | CCIS and Local EC: Comité de Ética del Instituto Medico Platense (CEDIMP) Boulevard 51 N°335, La Plata, Buenos Aires, Argentina | Dr. Gustavo J. Chaparro |
| 005787 | CCIS and Local EC: CEI CEMER - Comité de Ética en Investigación de CEMER Esmeralda 1550 (B1602DQD), Florida, Vicente López, Buenos Aires, Argentina | Dr. Maria de los Angeles Harris |
| 005790 | Comité de Ética Independiente Consultorios Integrados CEICI Italia 424, Rosario ZC 2000, Santa Fé, Argentina | Lic. Javier Alejandro Bilbao |
| 005792 | Comité de Ética en Investigacion Clínica (CEIC) Larrea 1381 3°"A", Ciudad Autónoma de Buenos Aires, Buenos Aires, Argentina | Dr. Diego Hernan Fridman |
| 005836 | Comité de Ética en Investigación (CEI) del Centro de Investigaciónes Metabólicas, Viamonte 2278 C1056ABJ, CABA, Argentina | Dra. Roxana Secundini |
| 005844 | Comité de Ética en Farmacología Clínica de la Fundación CIDEA -CEFC Paraguay 2041 piso 9°H (C1121ABE) - CABA, Argentina | Dr. Claudio F. Bargas Modernell |
| 005871 | Comité de Ética para Investigación Clínica "Fundación Dr J R Villavicencio" Alvear 854 –S2000, Rosario, Santa Fé, Argentina | Dr. Carlos Lovesio |
| 005872 | Comité de ética Independiente Patagónico, Urquiza 646, Santa Rosa (6300), La Pampa | Saulo Cortes |
| 005873, 005874 | FumeLit (Fundación Médica del Litoral) Av Freyre 3048 -3000, Santa Fé | Dr. Guillermo Heredia |
| 005886 | Comité Independiente de Ética en Investigación Biomédica de la Fundación Neurológica Argentina Uruguay 824, piso 1, C1015ABR. Ciudad Autónoma de Buenos Aires, Argentina | Dr. Mariana Bendersky |
| **Australia** |  |  |
| 005251, 005311, 005714, 005255 | Belberry Ltd, 129 Glen Osmond Rd, Eastwood SA 5063 | Barry Chatterton |
| 005252, 005253, 005254, 005255, 005256, 005307, 005452, 005461, 005476 | Western Sydney Local Health District HREC, Research Office, Level 2, REN Building, Westmead Hospital, Hawkesbury & Darcy Roads Westmead NSW 2145 | Dr. Tony Skapetis |
| **Austria** |  |  |
| 005257, 005313, 005348, 005349, 005350, 005351, 005407, 005725 | Ethikkommission für das Bundesland, Salzburg, Sebastian-Stief-Gasse 2, 5020 Salzburg | Mag. Dr. Alexander Hönel |
| **Belgium** |  |  |
| 005258, 005259, 005260, 005261, 005408, 005409, 005480, 005542, 005585, 005597, 005830 | Cliniques Universitaires Saint-Luc UCL Bruxelles, Comité d'Ethique Hospitalo-Facultaire Saint-Luc - UCL - OM 003 | Prof. Jean-Marie Maloteaux |
| **Canada** |  |  |
| 005271, 005273, 005276, 005277, 005278, 005356, 005357, 005503, 005504, 005505, 005586, 005619, 005620, 005721, 005722, 005893, 005894, 005978, 005979 | Advarra d/b/a Schulman Associates Institutional Review Board, Inc, 4445 Lake Forest Drive Suite 300, Cincinnati Ohio 45242 | Sharon Lynn Nelson, M.S.N, RN, C.N.S. |
| 005272 | Nova Scotia Health Authority Research Ethics Board (NSHA REB) 5790 University Avenu Room 118 CCR Building Halifax, Nova Scotia B3H 1V7 902-470-6511 | Dr. Chris MacKnight |
| 005274 | Concordia Hospital Ethics Committee Sunil Desai, MD (Acting Chair) 1095 Concordia Avenue Winnipeg, MB, Canada R2K3S8 204-661-7160 | Valerie Wiebe |
| 005275, 005720 | Health Research Ethics Board of Alberta Clinical Trials Committee1500, 10104 - 103 Avenue NW, Edmonton, Alberta T5J 4A7 877-423-5727 | Sunil Desai, MD |
| 005719, 005781 | IUCPQ Research Ethics Committee Institut universitaire de cardiologie et de pneumologie de Québec – Université Laval 2725, chemin Sainte-Foy Québec (Québec) Canada G1V 4G5 418-656-8711 | Jamila Chakir, PhD |
| 005411 | University of Saskatchewan Research Ethics Board (REB) or Committee1607 - 110 Gymnasium Place, S7N 0W9 306-966-2975 | Beth Davis, PhD |
| 005782 | Hamilton Integrated Research Board, 293 Wellington Street North, suite 102, Hamilton, Ontario L8L 8E7, 905-521-2100 X42013 | Dr. Mark Inman, MD, PhD |
| **Chile** |  |  |
| 005279 | Comité Ético Científico del Servicio de Salud Metropolitano Sur OrienteAvenida Concha y Toro 3459 Puente Alto, Santiago, | Dr. Patricio Michaud |
| 005360, 005600, 005880 | Comité de Ética Cientfífico del Servicio de Salud Metropolitano SurAvenida Santa Rosa 3453. San Miguel, Santiago, | Verónica Rivera S. |
| 005454, 005455, 005483, 005599, 005769, 005879, 005941 | Comité de Ética Científico del Servicio de Salud Metropolitano OrienteAvenida Salvador 364 Providencia, Santiago, | Dr. Sara Chernilo |
| 005598, 005601 | Comité Etico Científico del Servicio de Salud Viña del Mar - QuillotaCalle Limache 1307 Viña del Mar, Valparaíso, | Dr. Héctor Camerati Villar |
| 005712 | Comité Ético Científico, Servicio de Salud Valparaíso-San Antonio | Dr. Rodrigo/Vergara Fischer |
| 005770 | Comité Ético Científico, Servicio de Salud Concepción | Dr. Nelson Pérez Terán |
| **China** |  |  |
| 005727 | China-Japan Friendship Hospital Medicine/Medical device clinical trial Ethics Committee, No. 2 East Sakura Road, Heping li, Chaoyang District, Beijing, China | Chen Yanfen |
| 005728 | Medical Ethics Committee of Hebei General Hospital, Room 623, 6th floor, Outpatient building, Hebei General Hospital, No.348 Heping West Road, Shijiazhuang, Hebei Province, China | Shuhui Zhang |
| 005729 | The First Affiliated Hospital of Soochow University Ethics Committee, No.188, Shizi Street, Suzhou, Jiangsu Province, China | Zhou Wu |
| 005730 | Ethics Committee of Beijing Chao-yang Hospital, Capital Medical University, Room 307, West Yard, Beijing Chao-Yang Hospital, Beijing, China | Lihua Tian |
| 005731 | Ethics Committee of Beijing Friendship Hospital, Capital Medical University, No.95 Yongan Road, Xicheng District, Beijing, China | Yang Chunxiu |
| 005732 | Ethics Committee on drug clinical trials of The Second Hospital of Hebei Medical University, No.215, Heping west Road, Shijiazhuan city, Hebei Province, China | Zhao Yile  Sun Qian |
| 005733 | Ethics Committee of Beijing Anzhen Hospital, No. 2 Anzhen Road, Chaoyang District, Beijing, China | Yang Kexu  Suo Wei |
| 005736 | Anhui province hospital clinic research ethic committee, No. 17 Lujiang Road, Luyang District, Hefei City, Anhui Province, China | Chen Shengnan |
| 005737 | Huadong Hospital Affiliated to Fudan University, Ethics Committee, No.221,Yan'an West Road, jing'an District, Shanghai, China | Sha Yinghao |
| 005738 | Ethics Committee of Qinghai Provincial People's Hospital, The second floor of the complex building, No.2, Gonghe Road, Chengdong Distric, Xi'ning City, Qinghai Province, China | Zhang Jingjing |
| 005740 | EC of Central Hospital of Minhang District, No. 170, Xinsong Road. Shanghai, China | Mo Yanqing |
| 005742 | Ethics Committee for Institutional Review of Affiliated Hospital of Guangdong Medical College, 57 Renmin Avenue, Zhanjiang City, Guangdong Province, China | Mingyi LiNA |
| 005743 | Ethics Committee of The Affiliated Hospital of Xuzhou Medical College, 99 Huaihai West Road, Quanshan District, Xuzhou, Jiangsu Province, China | Zhuming Shao Haijing Jiang |
| 005745 | Drug clinical Trial Ethics Committee of Guizhou Provincial People's Hospital, No. 83, East Zhongshan Road, Guiyang, Guizhou Province, China | Lei Luo |
| 005746 | EC of Sir Run Shawn Hospital School of Medicine, Zhejiang University, N0.3 East Qingchun Road, Hangzhou, Zhejiang Province, China | Xu Ming |
| 005748 | Ethics Committee of the First Hospital of Lanzhou University, The second floor of the bachelor apartment, No1, Donggang West Road, Chengguan District, Lanzhou City, Gansu Province, China | Yao Jia |
| 005750 | Medical Ethics Committee of Affiliated Hospital of Guizhou Medical University, 10th floor, North Campus of Guizhou Medical University, No. 9, Beijing Road, Guiyang Province, China | Jian Liu |
| 005751 | Drug Clinical Trial Ethics Committee of Shenzhen People' s Hospital No.1017, Dongmen North Road, Shenzhen City, Guangdong Province, China | Zheng Xuefen |
| 005752 | Ethics Committee of The Second People's Hospital of Shenzhen No.3002, Sungang West Road, Futian District,Shenzhen City, Guangdong Province, China | Wenpeng Guo |
| 005753 | Ethics Committee on drug clinical trials of The First Affiliated Hospital of Baotou Medical College Inner Mongolia University of Science and Technology, No.41, Linyin Road, Kundulun District, Baotou city, The Inner Mongolia Autonomous region Province, China | Chen Congcong |
| 005754 | Biomedical Ethics Committee of Haikou People's Hospital, 43 Renmin Avenue, Haikou City, China | Qiu Yinglin |
| 005755 | Ethics committee of Xiangya Hospital Central South University, No.87, Xiangya Road, Kaifu District, Changsha, Hunan Province, China | Pingsheng Xu |
| 005756 | Ethics Committee of Guangzhou Red Cross Hospital, No.396, Tongfuzhong Road, Haizhu District, Guangzhou City, Guangdong Province, China | Peng Fen |
| 005757 | Medical Ethics Committee of the Second Affiliated Hospital of Nanchang University, 10th floor, Zonghe Building, No. 1, Minde Road, Nanchang, Jiangxi Province, China | Yang Yazhi |
| 005758 | Ethics Committee of The Third Hospital of Hebei Medical University Room 281, Research building, The Third Hospital of Hebei Medical University, 139 Ziqiang Road, Qiaoxi District, Shijiazhuang City, Hebei Province, China | Zhang Chunhuan |
| 005760 | Drug Clinical Trial Ethics Committee of Yanbian University Hospital, Room 310, Administrative building, No. 1327, Juzi Street, Yanji City, Jinlin Province, China | Xu Xiangshu |
| 005764 | Ethics Committee of the First Hospital of Changsha, No. 311 Yingpan Road, Kaifu District, Changsha, Hunan Province | He Gefei |
| 005765 | Ethics Committee of Nanjing First Hospital, 68 Changle Road, Qinhuai District, Nanjing City, Jiangsu Province, China | Zhou Jie |
| 005766 | EC of The Second Affiliated Hospital of Nanjing Medical University, No.121 Jiangjiayuan, Gulou District, Nanjing City, Jiangsu Province, China | Zhang Juan |
| 005767 | EC of Hangzhou First People's Hospital, No. 261 Huansha Road, Shangcheng District, Hangzhou, Jiangsu Province, China | Lu Yun |
| 005768 | Clinical Trial Ethics Sub-Committee, West China Hospital, Sichuan University Room 412, old No. 8 Teacing Building, West China Hospital, Sichuan University, No.37 Guoxue Lane, Wuhou District, Chengdu, Sichuan Province, China | Li Na  Zuo Zejin |
| 005777 | Biomedical Research Ethics Committee of Peking University First Hospital  No.8 Xishiku Avenue, Xicheng District, Beijing, China | Yu Rong Hui  Jiang Jie |
| 005810 | Ethics Committee of the First Hospital of China Medical University, Office of Ethics Committee, 11th floor, Building 1, No. 92 North Second Road, Heping Dstrict, Shenyang, Liaoning Province, China | Zhang Caixia |
| 005811 | Ethics Committee of the first hospital of China Medical University, 151 Li Bing Road, Zhangjang Hi-Tech Park, Shanghi 201203, China | Wang Yaqi |
| 005812 | EC of Jiangsu Province Hospital, NO.300 Guangzhou Road, Gulou District, Nanjing, Jiangsu Province, China | Jun Zhao |
| 005813 | EC of The First Affiliated Hospital, College of Medicine, Zhejiang University, No.79, Qingchun Road, Uptown District, Hangzhou, Zhejiang Province, China | Hua Feiyang |
| 005814 | EC of Jiangxi Pingxiang People's Hospital  No.8 Zhongda Road, Wugong Mountain Development Area, Pingxiang City, Hunan Province, China | Tong Xiangxia |
| 005816 | Ethics Committee for The First Affiliated Hospital of Guangxi Medical University, No. 6 Shuangyong Road, Nanning City, Guangxi Province, China | Yanwen Zhou |
| 005817 | Ethics Committee of the First People's Hospital of Changde, The second floor of corridor of No.2 and No.3 inpatient bulidng, No.818, Renmin Road, Wuling District, Changde City, Hunan Province, China | Jiming Wu |
| 005818 | Clinical Drug-Device and New Medical Technologies Ethics Committee of The first Affiliated Hospital, Sun Yat-sen university, No.58, Zhongshan 2nd Road, Yuexiu District, Guangzhou City, Guangdong Province, China | Li Ying Huang Shushan |
| 005887 | Ethics Committee of Wuhan Forth Hospital, 473 Hanzheng Street, Qiekou District, Wuhan City, China | Gao Zhaohun |
| 005897 | Ethics Committee of Changsha Central Hospital, Room 205 of the pharmaceutical sciences building, No.161, Shaoshannan Road, Yuhua District, Changsha City, Hunan Province, China | Mao Yanmei  Wu Yajuan |
| 005898 | Ethics Committee of Xiangtan Central hospital clinical drug trial, The fifth floor of outpatient building, No.120, Heping Road, Xiangtan City, Hunan Province, China | Liu Chan |
| 005899 | EC of Shanghai East Hospital, Tongji University, No. 1800, Yuntai Road. Pudong District. Shanghai, China | Qinchuan Li |
| 005900 | Ethics Committee of The Affiliated Hospital of Inner Magnolia Medical University, No. 1 North Road, Huimin District, Hohhot, Inner Mongolia, China | Mingxing Hou |
| 005901 | EC of Taizhou Hospital of Zhejiang Province, No. 150. Ximen Road. Linhai City, Zhejiang Province, China | Aixiao Xia |
| 005902 | Ethics Committee of Inner Mongolia People’s Hospital, No. 20 Zhaowuda Road, Hohhot, Inner Mongolia, China | Dejun Sun |
| 005907 | Clinical Research and Applied Ethics Committee of The Third Affiliated Hospital of Guangzhou Medical University ,No.63, Duobao Road, Liwan District, Guangzhou City, Guangdong Province, China | Qixia Lliao |
| **Czech Republic** |  |  |
| 005412, 005456, 005457, 005458, 005484, 005544, 005545, 005546, 005560, 005561, 005562, 005570 | Etická komise, Fakultní nemocnice Královské Vinohrady, Šrobárova 1150/50, Praha 10, 100 34, Czech Republic | Prof. MUDr. Jan Pachl, CSc |
| 005413 | CEC, Etická komise, Fakultní nemocnice Královské Vinohrady, Šrobárova 1150/50, Praha 10, 100 34, Czech Republic  LEC, Lokální etická komise Nemocnice Mělník, Pražská 528, 276 01 Mělník, Czech Republic | MUDr. Zdeňka Hradecká |
| 005460 | CEC, Etická komise, Fakultní nemocnice Královské Vinohrady, Šrobárova 1150/50, Praha 10, 100 34, Czech Republic  LEC, Etická komise, Krajská nemocnice Liberec, a.s., Husova 10, 460 63, Liberec 1, Czech Republic | MUDr. Pavel Kočí |
| 005543 | CEC, Etická komise, Fakultní nemocnice Královské Vinohrady, Šrobárova 1150/50, Praha 10, 100 34, Czech Republic  LEC, Etická komise, Nemocnice Rudolfa a Stefanie Benešov, a.s., nemocnice Středočeského kraje, Máchova 400, 256 30 Benešov, Czech Republic | MUDr. Jaroslav Kraus, PhD |
| **France** |  |  |
| 005280, 005281, 005282, 005283, 005414, 005485 | CPP Sud Est III, Groupement Hospitalier Est, Bâtiment Pinel 59 Boulevard de Pinel, 69 500 Bron, France | François Chapuis |
| **Germany** |  |  |
| 005361-005381, 005415-005429, 005461-005469, 005486-005488, 005490-005493, 005563, 005572, 005573, 005578, 005579, 005587, 005588, 005602, 005603, 005605, 005799-005806, 005821, 005822, 005824, 005825, 005828, 005829, 005833, 005837, 005840, 005841, 005845, 005846, 005852-005856, 005940 | Ethikkommission der Medizinischen Hochschule Hannover, Carl-Neuberg-Str. 1, 30625 Hannover, Germany | Prof. Dr. Stefan Engeli |
| **Hungary** |  |  |
| 005430, 005431, 005432, 005433, 005434, 005435, 005436, 005437, 005438, 005494, 005495, 005496, 005547, 005548, 005564 | CEC , Medical Research Council Ethics Committee for Clinical Pharmacology (MRC-ECCP) /Egészségügyi Tudományos Tanács Klinikai Farmakológiai Etikai Bizottsága (ETT-KFEB), 1051 Budapest, Arany János u. 6-8., Hungary | Dr. Fürst Zsuzsanna |
| **Italy** |  |  |
| 005284 | CEC, Comitato Etico Milano Area B, Fondazione IRCCS Ca’ Granda Ospedale Maggiore Policlinico Via Francesco Sforza, 28 20122 Milano, Italy  LEC, Comitato Etico, Milano Area 2 Via F. Sforza n. 28, 20122 Milano | Dr. Gaetana Musarra |
| 005286 | CEC, Comitato Etico Milano Area B, Fondazione IRCCS Ca’ Granda Ospedale Maggiore Policlinico Via Francesco Sforza, 28 20122 Milano, Italy  LEC, Comitato Etico Regione Toscana , Area Vasta Nord Ovest Via Roma 67, 56126 Pisa | Dr. Romano Danesi |
| 005287 | CEC, Comitato Etico Milano Area B, Fondazione IRCCS Ca’ Granda Ospedale Maggiore Policlinico Via Francesco Sforza, 28 20122 Milano, Italy  LEC, Viale del Policlinico 155, 00161 Roma | Dr. Romano Danesi |
| 005383 | CEC, Comitato Etico Milano Area B, Fondazione IRCCS Ca’ Granda Ospedale Maggiore Policlinico Via Francesco Sforza, 28 20122 Milano, Italy  LEC, Comitato Etico Milano Area 1via G.B. Grassi, 74, 20157 MILANO | Prof.ssa Anna Maria Di Giulio |
| 005384 | CEC, Comitato Etico Milano Area B, Fondazione IRCCS Ca’ Granda Ospedale Maggiore Policlinico Via Francesco Sforza, 28 20122 Milano, Italy  LEC, Comitato Etico Seconda Università, degli Studi di NapoliOspedale dei Colli, Via Leonardo Bianchi snc, 80131 Napoli | Dr. Liberato Berrino |
| 005385 | CEC, Comitato Etico Milano Area B, Fondazione IRCCS Ca’ Granda Ospedale Maggiore Policlinico Via Francesco Sforza, 28 20122 Milano, Italy  LEC, Comitato Etico Area 1, AOU Ospedali Riuniti FoggiaViale Luigi Pinto, 1, 71122 Foggia | Prof. Ina Fischetti |
| 005386 | CEC, Comitato Etico Milano Area B, Fondazione IRCCS Ca’ Granda Ospedale Maggiore Policlinico Via Francesco Sforza, 28 20122 Milano, Italy  LEC, Comitato Etico Interaziendale della, Provincia di MessinaAOU Policlinico “G. Martino”, Via Consolare Valeria n. 1, 98125, Messina | Prof. Edoardo Spina |
| 005387 | CEC, Comitato Etico Milano Area B, Fondazione IRCCS Ca’ Granda Ospedale Maggiore Policlinico Via Francesco Sforza, 28 20122 Milano, Italy  LEC, Comitato Etico dell'Università "Sapienza", Viale del Policlinico 155, 00161 Roma | Prof. Giovanni Spera |
| 005388 | CEC, Comitato Etico Milano Area B, Fondazione IRCCS Ca’ Granda Ospedale Maggiore Policlinico Via Francesco Sforza, 28 20122 Milano, Italy  LEC, Comitato Etico dell'Università, Campus Bio Medico di Roma Via Álvaro del Portillo, 200, 00128 Roma | Prof. Claudio Buoni |
| 005389 | CEC, Comitato Etico Milano Area B, Fondazione IRCCS Ca’ Granda Ospedale Maggiore Policlinico Via Francesco Sforza, 28 20122 Milano, Italy  LEC, Comitato Etico Palermo 1Via del Vespro 129, 90127 Palermo | Dr. Salvatore Leone |
| 005439 | CEC, Comitato Etico Milano Area B, Fondazione IRCCS Ca’ Granda Ospedale Maggiore Policlinico Via Francesco Sforza, 28 20122 Milano, Italy  LEC, Comitato Etico di Area, Vasta Emilia CentroVia Albertoni, 15, 40138 Bologna | Dr. Primiano Iannone |
| 005470 | CEC, Comitato Etico Milano Area B, Fondazione IRCCS Ca’ Granda Ospedale Maggiore Policlinico Via Francesco Sforza, 28 20122 Milano, Italy  LEC, Comitato Etico, della LiguriaLargo Rosanna Benzi 10, 16132 Genova | Lawyer Paolo Gianatti |
| 005471 | CEC, Comitato Etico Milano Area B, Fondazione IRCCS Ca’ Granda Ospedale Maggiore Policlinico Via Francesco Sforza, 28 20122 Milano, Italy  LEC, Comitato Etico Campania SudVia Marconi, 66, 80049 - Torre del Greco (NA) | Dr. Celestino Todisco |
| 005472 | CEC, Comitato Etico Milano Area B, Fondazione IRCCS Ca’ Granda Ospedale Maggiore Policlinico Via Francesco Sforza, 28 20122 Milano, Italy  LEC, Comitato Etico Area Vasta Emilia NordPoliclinico di Modena, Via Largo del Pozzo 7, 41124, Modena | Prof. Sebastiano Calandra  Buonaura |
| 005473 | CEC, Comitato Etico Milano Area B, Fondazione IRCCS Ca’ Granda Ospedale Maggiore Policlinico Via Francesco Sforza, 28 20122 Milano, Italy  LEC, Comiatto Etico Milano Area 3Piazza Ospedale Maggiore, 3, 20162 Milano | Dr. Paolo Rossi |
| 005474 | CEC, Comitato Etico Milano Area B, Fondazione IRCCS Ca’ Granda Ospedale Maggiore Policlinico Via Francesco Sforza, 28 20122 Milano, Italy  LEC, Comitato Etico, Istituti Clinici Scientifici Maugeria Spa - IRCCSVIA Salvatore Maugeri, 4, 7100 Pavia | Dr. Liberato Berrino |
| 005497 | CEC, Comitato Etico Milano Area B, Fondazione IRCCS Ca’ Granda Ospedale Maggiore Policlinico Via Francesco Sforza, 28 20122 Milano, Italy  LEC, Comitato Etico Area, Vasta Emilia NordSegreteria Locale di Parma, Azienda Ospedaliero-Universitaria di Parma, Via Gramsci 14 43126 PARMA | Prof. Sebastiano Calandra Buonaura |
| 005574 | CEC, Comitato Etico Milano Area B, Fondazione IRCCS Ca’ Granda Ospedale Maggiore Policlinico Via Francesco Sforza, 28 20122 Milano, Italy  LEC, Comitato Etico indipendentePoliclinico Tor Vergata, Viale Oxford, 81 - 00133 Roma | Dr. Maria Grazia Marciani |
| 005723 | CEC, Comitato Etico Milano Area B, Fondazione IRCCS Ca’ Granda Ospedale Maggiore Policlinico Via Francesco Sforza, 28 20122 Milano, Italy  LEC, Comitato Etico Unico Regionale, - C.E.U.R.c/o IRCCS Centro di Riferimento Oncologico C.R.O., via Franco Gallini, 2 - 33081 AVIANO (PN) | Dr. Paolo Rossi |
| 005724 | CEC, Comitato Etico Milano Area B, Fondazione IRCCS Ca’ Granda Ospedale Maggiore Policlinico Via Francesco Sforza, 28 20122 Milano, Italy  LEC, Comitato Etico Seconda Università, degli Studi di NapoliOspedale dei Colli, Via Leonardo Bianchi snc, 80131 Napoli | Dr. Liberato Berrino |
| **Japan** |  |  |
| 005654 | 1-19-35, Hagiwara, Oita, 870-0921 | Keitaro Hashimoto  Akihiko Yura |
| 005509 | Nagata Hospital, 523-1, Shimomiyanagamachi, Yanagawa-shi, Fukuoka, 832-0059 | Eisuke Nagata  Yoshikatsu Nishi |
| 005511 | Sakaide City Hospital, 3-1-2, Kotobukicho, Sakaide-shi, Kagawa, 762-8550 | Teruhisa Taoka |
| 005656 | Kinki University Hospital, 377-2, Onohigashi, Osakasayama-shi, Osaka, 589-8511 | Hiroshi Ikegami  Tetsuya Mitsudomi |
| 005655 | Tosei General Hospital, 161, Nishioikawa-cho, Seto-shi, Aichi, 489-8642 | Masazumi Ajioka  Yasuhiro Kondo |
| 005815 | Showa University Hospital, 1-5-8, Hatanodai, Shinagawa-ku, Tokyo, 142-8666 | Masahiko Izumizaki |
| **Mexico** |  |  |
| 005641 | Mexico Centre For Clinical Research, S.A. de C.V., 709, Col. Del Valle, Del. Benito Juárez, Ciudad de México, C.P. 03100 | Alberto Warman Gryj. |
| 005642 | Comité de ética en Investigación del Instituto Jalisciense de Investigación Clínica S.A. de C.V., Calle Penitenciaria 20. Colonia Centro | Ricardo Osvaldo Jauregui Franco |
| 005643 | Comité de ética en Investigación del Instituto Jalisciense de Investigación Clínica S.A. de C.V., Calle Penitenciaria 20. Colonia Centro | Ricardo Osvaldo Jauregui Franco |
| 005644 | Unidad de Investigación Clínica en Medicina, S.C., Avenida de la Clinica no. 2520, Int. 520. Colonia Sertoma, C.P. 64718. Monterrey, nuevo León. | Salvador Valdovinos |
| 005646 | Mexico Centre For Clinical Research, S.A. de C.V., 709, Col. Del Valle, Del. Benito Juárez, Ciudad de México, C.P. 03100 | Alberto Warman Gryj. |
| 005647 | CEI Hospital La Misión S.A. de C.V., Avenida del Hospital 112, 1° y 2° piso, Col. Sertoma, C.P. 64718. Monterrey, nuevo León | Felipe ángel Robledo Padilla |
| **Netherlands** |  |  |
| 005289, 005290, 005390-005393, 005440, 005441, 005550, 005551, 005575, 005726, 005795, 005858, 005881 | METC Stichting BEBO, Stationsstraat 9, 9401 KV Assen, The Netherlands | J.R.B.J. Brouwers, PhD |
| **New Zealand** |  |  |
| 005291, 005292, 005293, 005294, 005295, 005296, 005442, 005693, 005702, 005783, 005784 | Health and Disability Ethics Committee, Ministry of Health, Freyberg Building 20 Aitken Building, PO BOX 5013 Wellington 6001 NZ | Dr. Brian Fergus |
| **Peru** |  |  |
| 005515, 005516, 005518, 005519, 005520, 005521, 005523, 005713, 005715, 005861, 005862, 005942 | Comité Institucional de Ética en Investigación de la Asociación Benéfica Prisma, Calle Carlos Gonzáles 251 San Miguel -15088 Lima, Peru | Salomón Zavala Sarrio |
| 005297, 005517 | Comité de ética en Investigación del Hospital Nacional Guillermo Almenara Irigoyen – ESSALUD, Av. Miguel Grau 800, La Victoria 15033, Peru | Demetrio Molero Castro |
| 005522 | Comité Institucional de Ética en la Investigación del Hospital Nacional Cayetano Heredia, Av. Honorio Delgado 430, Urb Ingeniería, San Martín de Porres, Lima | Jorge Luis Hung Yep |
| 005859 | Comité Institucional de Bioética de Via Libre, Paraguay 478, Cercado de Lima 15001, Peru | Lic. Karen Cruz Azaña |
| **Poland** |  |  |
| 005524, 005525, 005526, 005528, 005529, 005530, 005531, 005532, 005533, 005534, 005536, 005537, 005538, 005577 | Komisja Bioetyczna przy Śląskiej Izbie Lekarskiej, ul. Grażyńskiego 49a, 40-126 Katowice | Prof. Krystyn Sosada, MD, PhD |
| **Russia** |  |  |
| 005552 | Independent Interdisciplinary Committee on Ethics Evaluation of Clinical Trials, 20, build. 1, Delegatskaya str., 127473, Moscow, Russia  LEC at Federal State Institution "Federal Scientific Clinical Center of specialized kinds of medical care and medical technology of the Federal Medical and Biological Agency", 28, Orekhovy bulv., 115682, Moscow | Konstantin Inalovich Telbloev Danilevskaya Olesya Vasilevna |
| 005553 | Independent Interdisciplinary Committee on Ethics Evaluation of Clinical Trials, 20, build. 1, Delegatskaya str., 127473, Moscow, Russia  EC at Federal State Governmental Establishment, Burdenko Main Military Clinical Hospital of Russian Federation Defense Ministry, 3, Hospitalnaya square, 105229, Moscow | Sergey Alexandrovich Chernov |
| 005554 | Independent Interdisciplinary Committee on Ethics Evaluation of Clinical Trials, 20, build. 1, Delegatskaya str., 127473, Moscow, Russia  LIEC at SBHI of Leningrad Region, Gatchina Clinical Interdistrict Hospital, 15a, bld. 1, Roshchinskaya str., Gatchina, Leningrad Region, 188300, Russia | Vadim Ivanovich Kuzko |
| 005555 | Independent Interdisciplinary Committee on Ethics Evaluation of Clinical Trials, 20, build. 1, Delegatskaya str., 127473, Moscow, Russia  LEC at Non-governmental private Healthcare Institution, Scientific Clinical Center of Joint Stock Company Russian Railways, 20, Chasovaya str., 125315, Moscow | Vladimir Vasilyevich Popov |
| 005556 | Independent Interdisciplinary Committee on Ethics Evaluation of Clinical Trials, 20, build. 1, Delegatskaya str., 127473, Moscow, Russia  LEC at Municipal autonomous Institution City Clinical Hospital, #14 15B, 22 Partsyezda str., 620039, Ekaterinburg, Russia | Elena Vishneva |
| 005557 | Independent Interdisciplinary Committee on Ethics Evaluation of Clinical Trials, 20, build. 1, Delegatskaya str., 127473, Moscow, Russia  LEC at the State Budgetary Healthcare Institution of Moscow “City Clinical Hospital #51 of Healthcare Department of Moscow”, 7/33, Alyabyeva str., 121309, Moscow | Andrey Vladimirovich Chigirev |
| 005565 | Independent Interdisciplinary Committee on Ethics Evaluation of Clinical Trials, 20, build. 1, Delegatskaya str., 127473, Moscow, Russia  FGBU "Policlinika #5", 14, Plushchiha str, 119121, Moscow | Olga Vladimirovna Shishlacheva |
| 005566 | Independent Interdisciplinary Committee on Ethics Evaluation of Clinical Trials, 20, build. 1, Delegatskaya str., 127473, Moscow, Russia  PharmNadzor LLC3, Torzhkovskaya str., office 230, Saint-Petersburg, 197342, Russia | Alexey Yakovlevich Malikov |
| 005567 | Independent Interdisciplinary Committee on Ethics Evaluation of Clinical Trials, 20, build. 1, Delegatskaya str., 127473, Moscow, Russia  LEC at SBHI of Leningrad Region Occupational Pathology Center, 27, Lit. O, Mechnikov av., Saint-Petersburg, 195271, Russia | Vladimir Dmitrievich Balunov |
| 005568 | Independent Interdisciplinary Committee on Ethics Evaluation of Clinical Trials, 20, build. 1, Delegatskaya str., 127473, Moscow, Russia  EC at SBEI HPE "First Saint-Petersburg State Medical University named after I.P. Pavlov” of Ministry of Health of the Russian Federation at 10, Rentgena str., Saint-Petersburg, 197101, Russia | Edvin Eduardovich Zvartau |
| 005591 | Independent Interdisciplinary Committee on Ethics Evaluation of Clinical Trials, 20, build. 1, Delegatskaya str., 127473, Moscow, Russia  LEC at Pirogov Russian National Research Medical University, Legal Address: 1, Ostrovityanova str., 117997, Moscow | Ivan Gennadyevich Gordeev |
| 005592 | Independent Interdisciplinary Committee on Ethics Evaluation of Clinical Trials, 20, build. 1, Delegatskaya str., 127473, Moscow, Russia  LEC at FSBI «Policlinic №1» of Department for Presidential Affairs of the Russian Federation, 26/28, Sivtsev Vrazhek pereulok, Moscow, 119002, Russia | Viktor Vladimirovich Kniga |
| 005593 | Independent Interdisciplinary Committee on Ethics Evaluation of Clinical Trials, 20, build. 1, Delegatskaya str., 127473, Moscow, Russia  LEC at SFEI of HPT "I.M. Sechenov First Moscow State Medical University" of the Ministry of Health of the Russian Federation, 8, bld.2, Trubetskaya str., Moscow, 119991, Russia | Dmitri Alexeevich Balalykin |
| 005594 | Independent Interdisciplinary Committee on Ethics Evaluation of Clinical Trials, 20, build. 1, Delegatskaya str., 127473, Moscow, Russia  LEC First City Clinical Hospital named after E.E. Volosevich, 1, Suvorova str., 163001, Arkhangelsk | Svetlana Alexeevna Tyukina |
| 005595 | Independent Interdisciplinary Committee on Ethics Evaluation of Clinical Trials, 20, build. 1, Delegatskaya str., 127473, Moscow, Russia  EC at SBEI HPE First Saint-Petersburg State Medical University named after I.P. Pavlov of Ministry of Health of the Russian Federation at 10, Rentgena str., Saint-Petersburg, 197101, Russia | Edvin Eduardovich Zvartau |
| **Serbia** |  |  |
| 005298 | Ethics Committee of Serbia, Vojvode Stepe 458, 11000 Belgrade, Serbia  LEC Institute for Pulmonary disease Sremska Kamenica, Put Dr Goldmana 4, 21204 Sremska Kamenica, Serbia | Prof. Nevena Secen |
| 005299, 005394, 005396 | Ethics Committee of Serbia, Vojvode Stepe 458, 11000 Belgrade, Serbia  LEC Clinical Center of Serbia, Pasterova 2, 11000 Belgrade, Serbia | Prof. Andrija Bogdanovic |
| 005300 | Ethics Committee of Serbia, Vojvode Stepe 458, 11000 Belgrade, Serbia  LEC General Hospital Valjevo, Sindjeliceva 62, 14000 Valjevo, Serbia | Mira Vukovic, MD |
| 005301 | Ethics Committee of Serbia, Vojvode Stepe 458, 11000 Belgrade, Serbia  LEC CHC Zvezdra, Dimitrija Tucovica 131, 11000 Belgrade, Serbia | Prof. Nebojsa Despotovic |
| 005302, 005842, 005843 | Ethics Committee of Serbia Vojvode Stepe 458, 11000 Belgrade, Serbia  LEC Municipal Institute for Lung Diseases and Tuberculosis, Presevska 36, 11000 Belgrade Serbia | Jelena Menkovic, MD |
| 005303 | Ethics Committee of Serbia, Vojvode Stepe 458, 11000 Belgrade, Serbia  Kliničko bolnički centar ''Bežanijska Kosa'',  Clinical Hospital center ‘’Bezanijska Kosa’’  Bežanijska Kosa bb, 11080 Beograd, Serbia | Mirjana Cvetkovic, MD |
| 005305 | Ethics Committee of Serbia, Vojvode Stepe 458, 11000 Belgrade, Serbia  Institut za plućne bolesti Vojvodine  Institute for pulmonary diseases of Vojvodina  Put dr Goldmana 4, 21204 Sremska Kamenica, Serbia | Prof. Nevena Secen |
| 005395 | Ethics Committee of Serbia, Vojvode Stepe 458, 11000 Belgrade, Serbia  Klinički centar Niš  Clinical center Niš  Bulevar dr Zorana Djijdjica 48, 18000 Niš, Serbia | Prof. Steva Stanisic |
| 005475, 005835 | Ethics Committee of Serbia, Vojvode Stepe 458, 11000 Belgrade, Serbia  Klinički centar Kragujevac  (Clinical center Kragujevac)  Zmaj Jovina 30, 34000 Kragujevac, Serbia | Ass. Prof Dejana Ruzic-Zecevic |
| **South Africa** |  |  |
| 005306, 005308, 005310, 005314, 005443, 005444, 005445, 005446, 005580, 005581, 005583, 005710, 005718, 005771, 005773, 005832, 005834, 005863, 005864, 005865, 005866, 005867, 005868, 005869, 005896 | Pharma-Ethics, 123 Amcor Road, Lyttleton Manor, Centurion, South Africa | Dr. C.S.J. Duvenage |
| 005309 | University of Stellenbosch HREC, 5ht Floor Teaching Block, Faculty of Medicine and Health Sciences, Francie van Zijl Drive, Parow, South Africa | Prof A. van der Merwe |
| 005312 | University of Cape Town HREC, Old Main Building, Groote Schuur Hospital, Floor E52, Room 23, Observatory, Cape Town, South Africa | Prof M. Blockman |
| **Spain** |  |  |
| 005328, 005329, 005330, 005331, 005332, 005334, 005335, 005336, 005338, 005400, 005401, 005402, 005451, 005498, 005558, 005559, 005584, 005596, 005774, 005775, 005776, 005809, 005823, 005870, 005884, 005885, 005888, 005889, 005890, 005891, 005960, 005961, 005962 | CEIC-Parc de Salut MAR, IMIM - Institut de Recerca Hospital del Mar, Parc de Recerca Biomèdica de Barcelona, C/ Dr. Aiguader, 88, 08003 Barcelona, Spain | Marta Guix Arnau |
| **Sweden** |  |  |
| 005403, 005404, 005539, 005589, 005606, 005607 | Etikprövningsmyndigheten, Box 2110, 750 02 Uppsala | Eva Lindeblad  Greger Lindberg |
| **Taiwan** |  |  |
| 005339 | CEC, Chang Gung Medical Foundation Institutional Review Board, No. 5, Fusing St., Gueishan Township, Taoyuan City, Taiwan  LEC, Research Ethics Review Committee, Far Eastern Memorial Hospital, No21. Nan-Ya S.R, Pan-Chiao, New-Taipei City, Taiwan | Shu-Wen Chang, MD |
| 005340 | CEC, Chang Gung Medical Foundation Institutional Review Board, No. 5, Fusing St., Gueishan Township, Taoyuan City, Taiwan  LEC, Institutional Review Board, Kaohsiung Medical University Chung-Ho Memorial Hospital, No. 100, Zuyu 1st Road, Kaohsiung City. | Jiaan-Der Wang, MD |
| 005341 | CEC, Chang Gung Medical Foundation Institutional Review Board, No. 5, Fusing St., Gueishan Township, Taoyuan City, Taiwan  LEC, Institutional Review Board, E-Da Hospital, No.6, Yida Road, Jiao-su Village, Yan-chao District, Kaohsiung City. | Chao Tien Hsu, MD |
| 005342 | CEC, Chang Gung Medical Foundation Institutional Review Board, No. 5, Fusing St., Gueishan Township, Taoyuan City, Taiwan  LEC, Taichung Veterans General Hospital Institutional Review Board, No. 1650 Taiwan Blvd sec. 4, Taichung City. | Jiaan-Der Wang, MD |
| 005343 | CEC, Chang Gung Medical Foundation Institutional Review Board, No. 5, Fusing St., Gueishan Township, Taoyuan City, Taiwan  LEC, Chang Gung Medical Foundation Institutional Review Board, 199 Tung Hwa North Road, Taipei City, Taiwan, 10507 | Tsang-Tang Hsieh, MD |
| 005344 | CEC, Chang Gung Medical Foundation Institutional Review Board, No. 5, Fusing St., Gueishan Township, Taoyuan City, Taiwan  LEC, Chang Gung Medical Foundation Institutional Review Board, 199 Tung Hwa North Road,Taipei City, Taiwan, 10507 | Tsang-Tang Hsieh, MD |
| 005540 | CEC, Chang Gung Medical Foundation Institutional Review Board, No. 5, Fusing St., Gueishan Township, Taoyuan City, Taiwan  LEC, Research Ethics Committee, China Medical University & Hospital, No.2 Yude Road, North Dist., Taichung City. | Martin M-T Fuh, MD |
| 005541 | CEC, Chang Gung Medical Foundation Institutional Review Board, No. 5, Fusing St., Gueishan Township, Taoyuan City, Taiwan  LEC, Research Ethics Committee, National Taiwan University Hospital, No 7, Chung Shan South Road, 100, Taipei, Taiwan | Daniel Fu-Chang Tsai, MD |
| 005807 | CEC, Chang Gung Medical Foundation Institutional Review Board, No. 5, Fusing St., Gueishan Township, Taoyuan City, Taiwan  LEC, Chang Gung Medical Foundation Institutional Review Board, 199 Tung Hwa North Road, Taipei City, Taiwan, 10507 | Tsang-Tang Hsieh, MD |
| **UK** |  |  |
| 005345, 005346, 005347, 005500, 005501, 005502, 005608, 005611, 005612, 005613, 005616, 005617, 005796, 005797, 005798, 005808, 005827, 005882, 005965, 005966, 005967, 005968 | East Midlands - Leicester South Research Ethics Committee, Royal Standard Place, Nottingham, NG1 6FS, United Kingdom | Mr John Aldridge |
| **United States** |  |  |
| 005001-005029, 005031-005086, 005088-005115, 005117-005124, 005126, 005128-005242, 005244-005250, 005648, 005650-005652, 005659-005671, 005673-005674, 005676-005692, 005694-005701, 005703-005709, 005903-005904, 005906, 005909-005913, 005915-005939, 005944-005945, 005947-005953, 005955-005957, 005969-005972, 005974-005976, 005983-005991, 005993-005997 | Advarra d/b/a Schulman Associates Institutional Review Board, Inc, 4445 Lake Forrest Drive, Suite 300, Cincinnati, OH 45242 | Sharon Lynn Nelson, MSN, RN, CNS |
| 005243 | Johns Hopkins Medicine Office of Human Subject Institutional Review Board  1620 McElderry Street, Reed Hall, Suite B-130, Baltimore, MD 21205-1911 | Joseph Carrese, MD |
